# A Pan-Cancer Bioinformatic Analysis of RAD51 Regarding the Values for Diagnosis, Prognosis, and Therapeutic Prediction

**DOI:** 10.3389/fonc.2022.858756

**Published:** 2022-03-10

**Authors:** Hengrui Liu, Jieling Weng

**Affiliations:** ^1^ Department of Pathology, The Second Affiliated Hospital of Guangzhou Medical University, Guangzhou, China; ^2^ Biocomma Limited, Shenzhen, China

**Keywords:** pan-cancer, RAD51, diagnosis, prognosis, therapeutic prediction

## Abstract

**Background:**

RAD51, a critical protein for DNA repairment, has been found to associate with multiple cancer types, but, so far, a systematic pan-cancer analysis of RAD51 has not been done yet.

**Methods:**

Data were obtained from multiple open databases and genetic alteration, gene expression, survival association, functional enrichment, stemness, mutation association, immunity association, and drug therapy association of RAD51were analyzed. A prognostic model of RAD51 for overall glioma was constructed as an example application of RAD51 as a biomarker.

**Results:**

RAD51 was overexpressed in 28 types of cancers and was associated with worse overall survival in 11 cancer types. RAD51 correlated genes were enriched in cell cycle terms. RAD51 was associated with cancer stemness, tumor mutational burden, and multiple immunomodulators in different cancer types. RAD51 expression was different across immune subtypes in 11 cancer types. RAD51 was closely associated with cancer immune microenvironments in some cancer types. Proliferating T cells was the cell type that expressed highest RAD51 across most of the cancer samples analyzed. RAD51 expression had an AUC of over 0.5 in 12 of the 23 ICB subcohorts. The Tumor Immune Dysfunction and Exclusion of 9 cancer types were different between RAD51 high and low groups. RAD51 expression showed negative correlations with the sensitivity of most drugs. A prognostic nomogram was constructed with a high confidence.

**Conclusion:**

RAD51 is a clinical valuable biomarker for multiple cancer types, regarding its potential power for diagnosis, prognosis, and therapeutic prediction.

## Introduction

Cancer is a major public health issue in the world. Affected by the COVID-19 pandemic, the diagnosis, and treatment of cancer were hampered and delayed, resulting in a short-term decrease in cancer incidence this year but might also lead to a potential increase in advanced-stage cancers and higher mortality in the next few years (1). Given the complexity of cancer development, there may be common mechanisms shared across different cancer types, hence, pan-cancer analysis of genes of interest, especially those genes that might play common roles in multiple cancer types, can contribute to clinical cancer diagnosis, prognosis, and therapies. The Cancer Genome Atlas (TCGA), Genotype-Tissue Expression (GTEx), and the Chinese Glioma Genome Atlas (CGGA) (2–4), as well as other available open databases, provide gene expression and clinical data of different cancer types, enabling pan-cancer analysis for understanding these genes across multiple cancer types.

Cancer arises from mutation. Genome instability and mutation have been thought to be a hallmark of cancer (5). In non-cancer cells, homologous recombination (HR) is essential for the maintenance of genome stability. HR repairs most DNA lesions through the complementarity of the DNA sequence. A critical step of the HR repairment is the binding of single-stranded DNA (ssDNA) to RAD51 protein near the repair sites (6, 7). This process has been found to be critical in the tumorigenesis of some cancer types. For instance, first found in breast cancer, the product of the breast cancer-associated gene 2 (BRCA2) mediates the chaperoning of RAD51 onto replication protein A (RPA)-coated ssDNA (8), thereby promoting cancer development. Therefore, HR-deficient in normal tissues has been suggested to be a potential mechanism for tumorigenesis (9).

An early clinical study showed that, for lung cancer patients, overexpression of RAD51 resulted in significantly worse survival (10), which inferred the potential prognostic value of RAD51 for cancer patients. Data suggested that the overexpression of RAD51 might promote cancer resistance to chemotherapy and radiotherapy (11–13). RAD51 was found to mediate the resistance of triple-negative breast cancer stem cells to the PARP Inhibitor (14). However, it remains unknown if the alteration in resistance results in the survival association of RAD51, but the bioinformatics study in the general prognostic power of RAD51 in some cancer types have been reported. For example, RAD51 was reported as prognostic biomarkers for colon cancer (15) and pancreatic cancer (16). In addition, data has suggested that RAD51 might associate with cancer immunity (17). In breast cancer and liver cancer, the role of RAD51 as a biomarker for immune cell infiltration has been reported (18, 19). However, so far, a systematic pan-cancer analysis of RAD51 has not been done yet. Therefore, this study aimed to investigate the clinical value of RAD51 for 33 cancer types, regarding the potential of RAD51 as diagnostic, prognostic, and immune therapy predictive biomarkers. The graphical abstracts were shown in **Supplementary Materials**.

## Methods

### RNA-Seq and Clinical Data Acquisition

Clinical and genomic data of glioma cohorts were downloaded from The Cancer Genome Atlas (TCGA), Genotype-Tissue Expression (GTEx), and the Chinese Glioma Genome Atlas (CGGA) in May 2021, in which the methods of acquisition and application complied with the guidelines and policies.

### Mutation Analysis

Mutation analyses were conducted using the cBioPortal (20) and the Open Targets Platform (21). The mutation or variant data were obtained from the TCGA PanCancer Atlas Studies and the UniProt. The 3D structure of the RAD51 protein was obtained from the RCSB PDB/PDB-101 [PDB 5nwl (22)].

### RNA-Seq Data Analysis and Plotting

All the analyses and plotting, including ROC plot, survival KM plot, nomogram construction, etc., were implemented by R foundation for statistical computing (2020) version 4.0.3 and ggplot2 (v3.3.2).

### Multiple Data Overexpression Analysis

Multiple data sets of the gene overexpression and DNA copy number gain of RAD51 were accessed and analyzed using the Oncomine (23).

### RAD51 Associated Genes Enrichment Analysis

Top 100 RAD51 correlated genes were identified using the GEPIA (24). The protein-protein interaction network of the top 100 RAD51 correlated genes was constructed using the STRING (25). The minimum required interaction score was set at the “high confidence” (>0.9). The active interaction source was set at “Experiments and Databases”. All the enrichment analyses were conducted using the Metascape (26).

### Immunofluorescence Staining of Cancer Cells

Immunofluorescence staining of the subcellular distribution of RAD51 within the nucleus, endoplasmic reticulum (ER), and microtubules of A431 squamous carcinoma cells, U-2 OS osteosarcoma cells, and GBM cells was obtained from the Human Protein Atlas (HPA) (27).

### Immunohistochemistry Staining

Immunohistochemistry staining images of RAD51 in cancer and non-cancer tissues were accessed from the HPA. Antibody HPA039310 was used to stain RAD51 except for stomach and stomach cancer (antibody CAB010381). The sample details and the general pathological annotations and results were provided by HPA.

### The Cell Cycle Association Analysis

Plots of single-cell RNA-sequencing data from the FUCCI U-2 osteosarcoma cell line were accessed and analyzed using the HPA. The temporal RAD51 mRNA expression patterns were characterized in individual cells using the Fluorescent Ubiquitination-based Cell Cycle Indicator (FUCCI) U-2 OS cell line.

### Stemness and Mutation Level Analysis

The OCLR algorithm was used to calculate the mRNAsi for the evaluation of cancer stemness. The tumor mutational burden (TMB) and microsatellite instability (MSI) were used to evaluate the mutation levels of samples.

### Immunomodulators Association

Immunomodulators association of RAD51 across cancer types were analyzed using TCGA data and the TISIDB (28).

### Immune Subtypes Association

Associations between RAD51 expression and immune subtypes across human cancers were analyzed using TCGA data and the TISIDB.

### Immune Cell Infiltration Analysis

The immune cell infiltration level was calculated using the TCGA cohort. The XCELL algorithms were used to estimate the immune cell infiltration levels (29).

### Single-Cell Sequencing Data Acquisition and Analysis

The single-cell data were accessed and analyzed using the TISCH (30).

### Immune Therapy Prediction

Immune checkpoint blockade (ICB) of RAD51 low (0-25%) and high (75-100%) groups were compared across multiple cancer types. Potential ICB response was predicted using the Tumor Immune Dysfunction and Exclusion (TIDE) algorithm (31). TCGA data were analyzed.

### Drug Sensitive Analysis

The GSCALite (32) was used to evaluate the area under the dose-response curve (AUC) values for drugs and gene expression profiles of RAD51 in different cancer cell lines. Drug sensitivity and gene expression profiling data of cancer cell lines in GDSC and CTRP were integrated for investigation. The Spearman correlation analysis was performed to analyze the association of expression of RAD51 and the small molecule drug sensitivity (IC_50_).

### Chemotherapy Prediction

The ROC plotter (33) was used to analyze associations of RAD51 transcriptome levels with therapeutic responses in breast cancer, ovarian cancer, glioma (female), and colon cancer (non-chemotherapy) cohorts.

### Statistical Analysis

Wilcox test or Kruskal-Wallis test was applied to compare gene expression differences. Kaplan-Meier analysis, log-rank test, and Cox regression test were used to conduct survival analysis. Pearson’s correlation test was conducted to evaluate the correlation of two variables except for the drug-sensitive analysis. A P<0.05 was considered to be statistically significant.

## Results

### Mutation of RAD51 in Cancers

**Figure 1:**
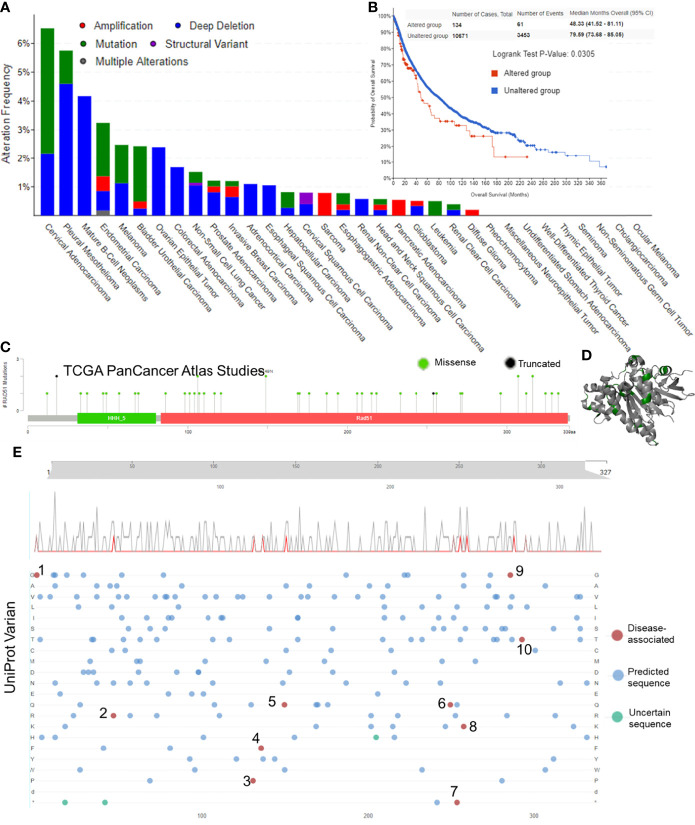
Gene alteration of RAD51 in cancers. **(A)** The RAD51 genetic alteration frequencies of different types of cancers. **(B)** The KM plot and log-rank analysis of the overall survival of cancer patients with or without RAD51 genetic alterations. **(C)** Mutation profile with the mutation frequencies of RAD51 in cancers. **(D)** The 3D structure of RAD51 protein with mutation sites (green) (PDB 5nwl). **(E)** Variants landscaped plot with mutated location and mutated amino acid. Top axis: amino acid position; gray curve: all mutations; red curve: disease-associated mutations; bottom plot: detailed location of the mutation (1, 2, 4, 6, 7, and 9: The disorder of congenital mirror movements; 3, 8, and 10: Fanconi anemia; 5: breast cancer).

The first section of this study was to explore whether RAD51 genetic alterations were associated with cancers. The alteration frequency bar plot showed that the total alteration frequencies of most of the cancer types were lower than 2.5%. Only cervical adenocarcinoma (6.52% of 46 cases), pleural mesothelioma (5.15% of 87 cases), mature B-Cell neoplasms (4.17% of 48 cases), and endometrial carcinoma (3.24% of 586 cases) had alteration frequencies of over 2.5%, but these cancer types had a relatively low case number except for the endometrial carcinoma (**Figure 1A**). Survival data suggested that altered RAD51 resulted in a worse overall survival in cancer patients, but the case number for altered groups was relatively small and the p-value was relatively large (**Figure 1B**). To further investigate the mutation of RAD51 in cancers, the TCGA mutation data was plotted and only 48 mutations were found, with 46 missense and 2 truncated mutations (**Figure 1C**), as shown in the 3D protein structure (**Figure 1D**). We also collected variant data from the Uniprot database and 10 disease-associated variants were found, but with only one variant associated with cancers (**Figure 1E**). Based on these results, this study suggested that, as an essential protein for the maintenance of genome stability, RAD51 had a low frequency of gene alterations. Thus, gene alterations of RAD51 might not be the major reason that drives cancers.

### The Diagnostic Value of RAD51 Across Cancers

**Figure 2:**
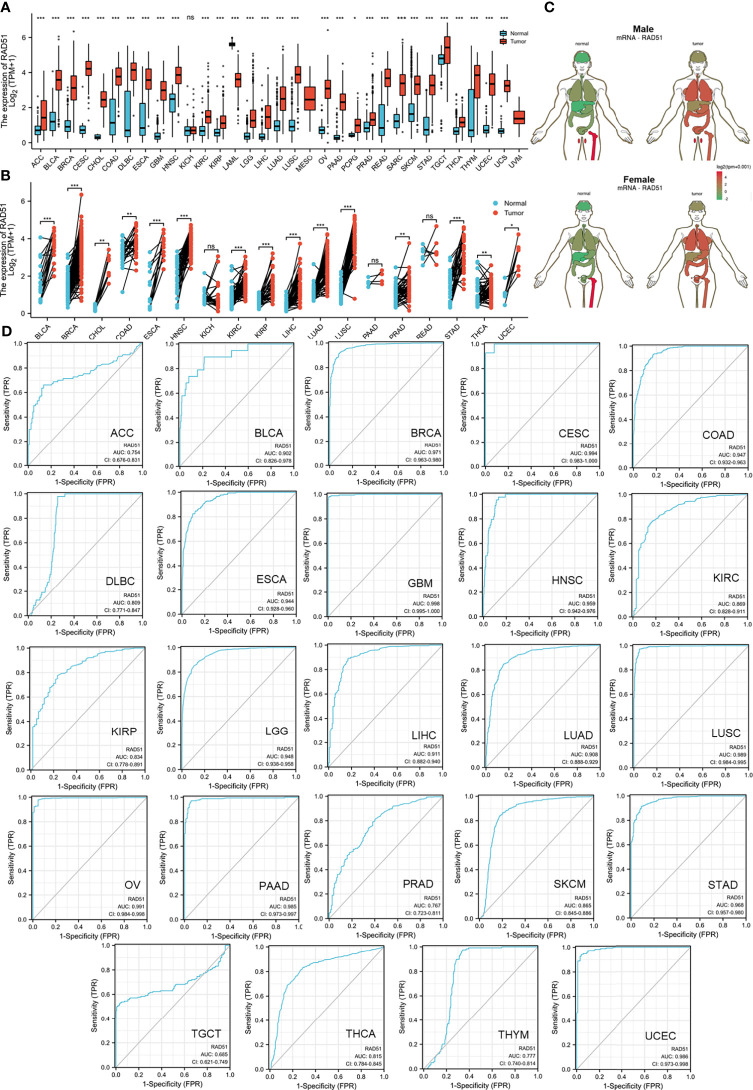
The diagnostic value of RAD51 in cancers. **(A)** The gene expression profile of RAD51 across all tumor samples and normal tissues. TCGA and GETx data were plotted. **(B)** Paired sample expression profile of RAD51 across all tumor samples and normal tissues. TCGA data were plotted. The full names of the cancer type abbreviations can be found in **S-Table 1**. **(C)** Anatomy plot of the gene expression profile of RAD51 across all tumor samples and paired normal tissues in females and males. **(D)** Diagnostic ROC of RAD51 in different cancer types. TCGA and GETx data were plotted. (ns, not significant; *P<0.05; **P<0.01; ***P<0.001).

This study hypothesized that although gene alterations of RAD51 might not be the major reason that drives cancers, the expression of RAD51 might associate with cancers. This study compared the expression of RAD51 across all tumor types and normal tissues using TCGA and GTEx data to determine the overexpression of RAD51 in cancers. A list of the cancer type abbreviations can be found in **S-Table 1**. Results showed that RAD51 was significantly overexpressed in 28 types of cancer. Mesothelioma (MESO) and uveal melanoma (UVM) have no comparable normal tissue, while acute myeloid leukemia (LAML) was the only cancer type that expressed lower RAD51 in cancer than in normal tissues (**Figure 2A**). To further compare cancer-noncancer at a better control, paired cancer noncancer samples from the same patients of available cancer types were also compared. Results showed that 15 cancer types were found to significantly overexpress RAD51 (**Figure 2B**). The anatomy plot of the gene expression profile of RAD51 across all tumor samples and paired normal tissues in females and males showed that RAD51 was overexpressed in cancer in most of the organs (**Figure 2C**). To evaluate the diagnostic value of RAD51 in these 28 types of cancers significantly overexpressing RAD51, diagnostic ROCs were plotted for these cancer types except for cholangiocarcinoma (CHOL), pheochromocytoma and paraganglioma (PCPG), and uterine carcinosarcoma (UCS) because of the low normal tissue numbers. Results showed that the AUCs of 15 cancer types were over 0.9. The AUCs of 5 cancer types were between 0.8-0.9. The AUCs of 3 cancer types were between 0.7-0.8. The AUC of testicular germ cell tumors (TGCT) was 0.685 (**Figure 2D**). These results suggested that RAD51 had an excellent diagnostic value for 18 cancer types (AUC>0.8), and had an acceptable diagnostic value for 3 cancer types (0.7<AUC<0.8).

### The Validation of the Diagnostic Value of RAD51 Across Cancers

**Figure 3:**
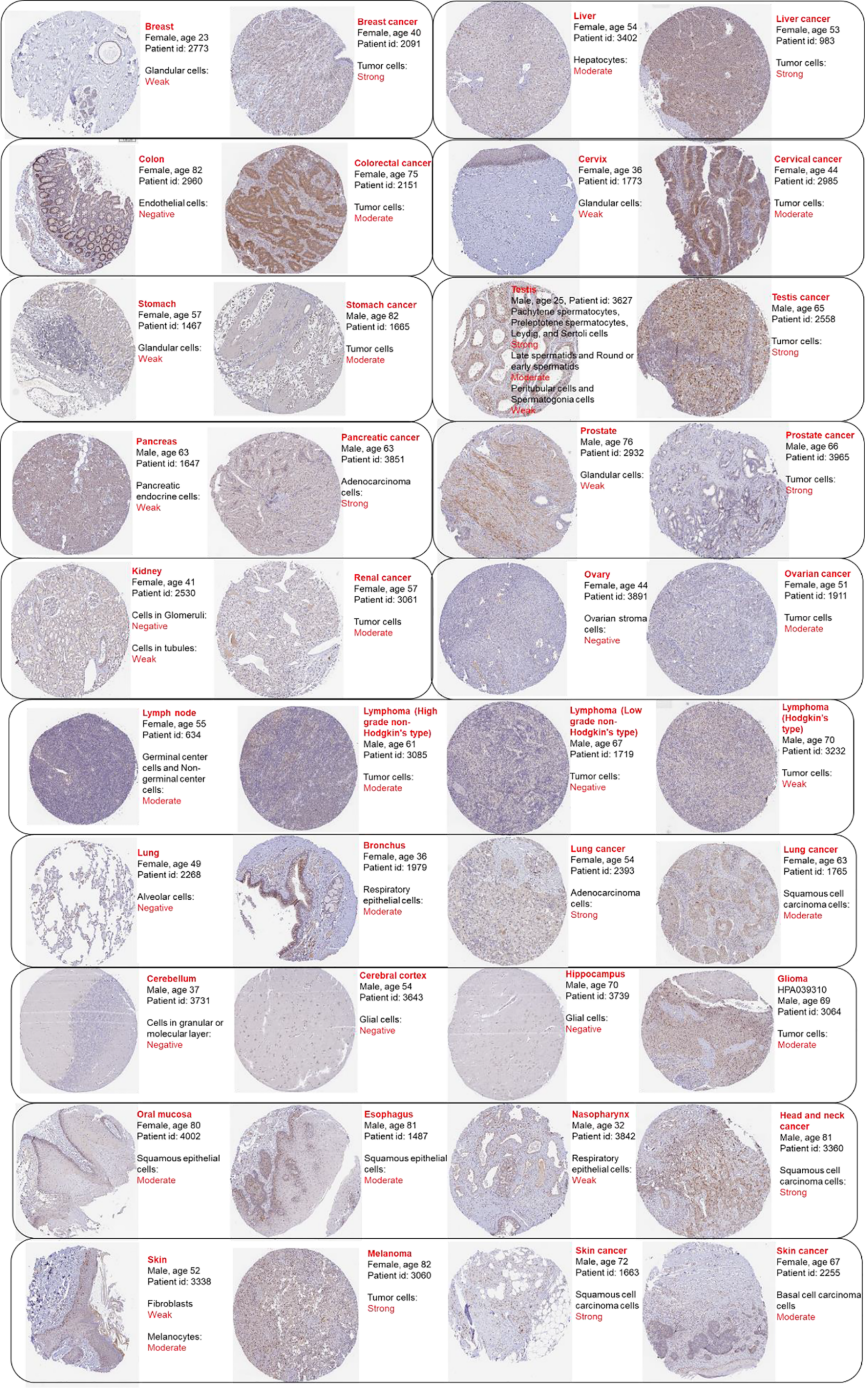
Representative protein staining images of RAD51 in cancer and normal tissues. The images were downloaded from the Human Protein Atlas (HPA). Antibody HPA039310 was used to stain RAD51 except for stomach and stomach cancer (antibody CAB010381). The sample details and the general pathological results were provided by HPA.

To validate the diagnostic value of RAD51 in cancers, the Oncomine was used to compare RAD51 expression or DNA copy number across analyses in different cancer types respectively. Results showed that bladder urothelial carcinoma (BLCA), breast invasive carcinoma (BRCA), cervical squamous cell carcinoma and endocervical adenocarcinoma (CESC), lymphoid neoplasm diffuse large B-cell lymphoma (DLBC), colon adenocarcinoma (COAD), esophageal carcinoma (ESCA), brain tumor (GBM&LGG), head and neck squamous cell carcinoma (HNSC), liver hepatocellular carcinoma (LIHC), ovarian serous cystadenocarcinoma (OV), lung cancer (LUAD&LUSC), prostate adenocarcinoma (PRAD), skin cutaneous melanoma (SKCM), and stomach adenocarcinoma (STAD) had significant RAD51 overexpression or RAD51 DNA copy number gain across 8, 18, 5, 11, 11, 7, 9, 13, 3, 5, 8, 6, 3, and 7 analyses respectively. Detailed analysis results and references were provided in **S-Figure 1**. These data validated the diagnostic value of RAD51 in most of the cancer types mentioned above. To investigate the expression of RAD51 at the protein level, we compared RAD51 protein staining of cancer and noncancer tissue across 15 tissue types. Representative images showed that 13 tissue types had a stronger staining signal of RAD51 in cancer than non-cancer tissues, including breast, liver, colon, cervix, stomach, pancreas, prostate, kidney, ovary, lung, glioma, head and neck cancer, and skin cancer and melanoma. Testis cancer had strong RAD51 staining but some cell components of the normal testis tissue also had strong signals. In the lymph node, high-grade non-Hodgkin’s type lymphoma had a strong RAD51 signal as normal lymph node, but low-grade non-Hodgkin’s type and Hodgkin’s type lymphoma had a weaker RAD51 signal (**Figure 3**). To notice, these RAD51 protein staining data were subjected to sample size, interindividual differences, and variations due to primary diseases, age, sex, etc, hence they were compared for reference only.

### The Prognostic Value of RAD51 Across Cancers

**Figure 4:**
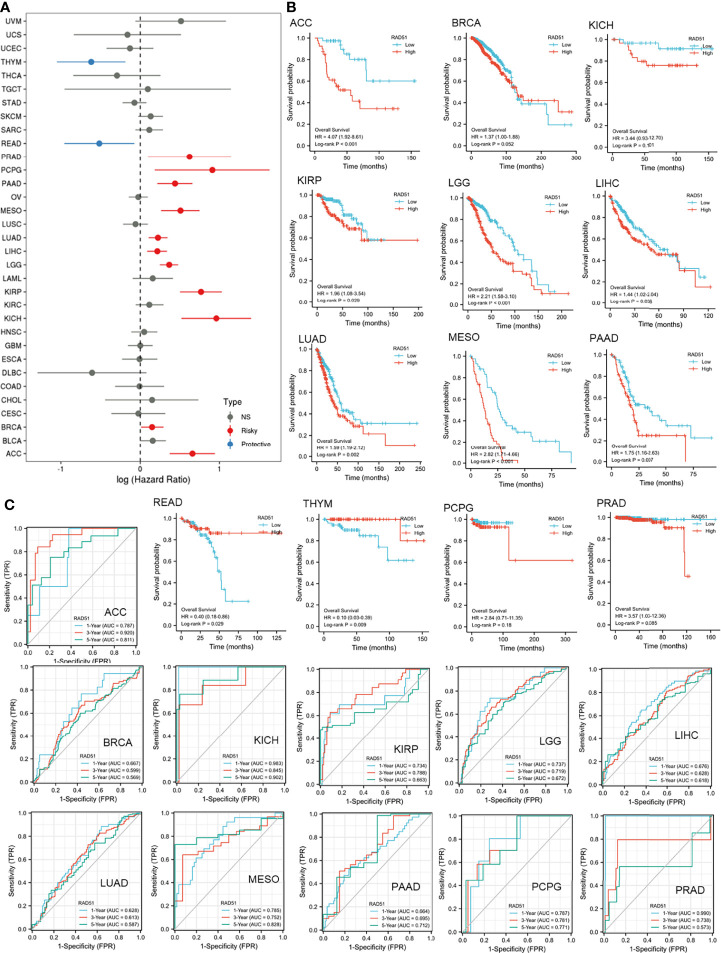
The prognostic value of RAD51 in cancers. **(A)** Univariate Cox regression analysis of RAD51 for overall survival in different cancer types. **(B)** The overall survival KM plot and log-rank analysis of high (50-100%) and low (0-50%) RAD51 patients of significant cancer types in Cox regression. **(C)** Time-dependent overall survival ROC of RAD51 in significantly risky cancer types in Cox regression (1-, 3-, and 5-year survival).

This study was also interested in the prognostic value of RAD51 in cancers. Thus, univariate overall survival Cox regression analysis of RAD51 was conducted across 33 cancer types. Results showed that RAD51 was significantly associated with worse overall survivals in 11 cancer types, while RAD51 was associated with better overall survivals in rectum adenocarcinoma (READ) and thymoma (THYM) (**Figure 4A**). KM plot and log-rank analysis of high (50-100%) and low (0-50%) RAD51 patients of significant cancer types in Cox regression were also used to further observe the association of RAD51 and the overall survival of patients. The KM plotting of high and low RAD51 patients showed that 9 cancer types remained significant in log-rank analysis (**Figure 4B**). To evaluate the prognostic value of RAD51 in the 11 types of cancers where RAD51 was significantly associated with worse overall survival, time-dependent prognostic ROCs were plotted. Results showed that the AUCs of adrenocortical carcinoma (ACC), kidney chromophobe (KICH), mesothelioma (MESO), and prostate adenocarcinoma (PRAD) were over 0.8 (at least for one of the prediction AUCs). AUCs of kidney renal papillary cell carcinoma (KIRP), brain lower-grade glioma (LGG), pancreatic adenocarcinoma (PAAD), and pheochromocytoma&paraganglioma (PCPG) were over 0.7 (at least for one of the prediction AUCs) (**Figure 4C**). These results suggested that RAD51 had an excellent prognostic value for the 5 cancer types (AUC>0.8), and had an acceptable prognostic value for the 4 cancer types (0.7<AUC<0.8).

### Potential Functions of RAD51 in Cancers

**Figure 5:**
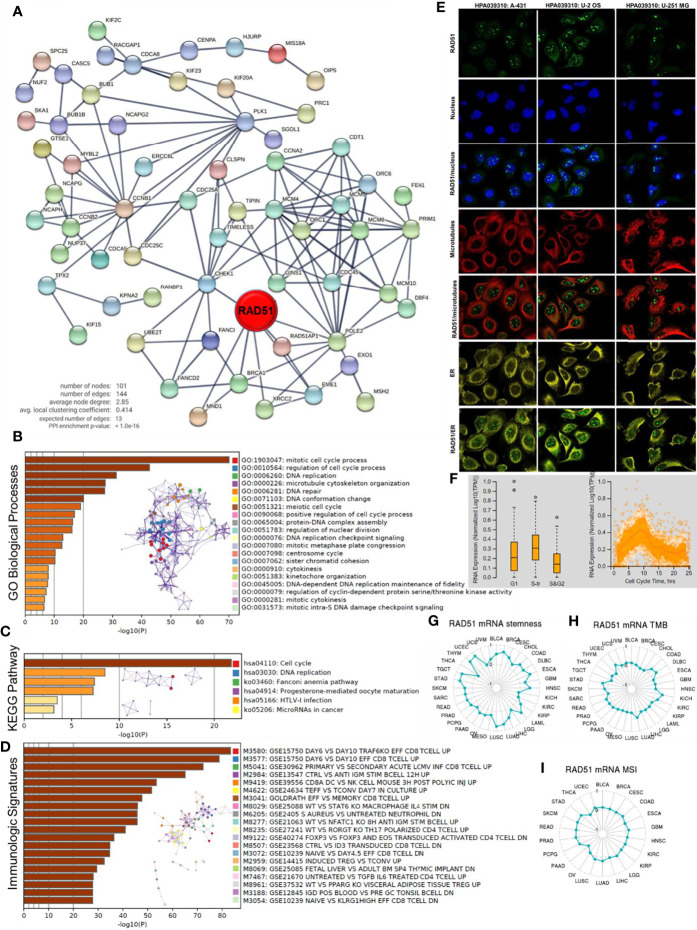
Potential functions of RAD51 in cancers. Top 100 RAD51 correlated genes were identified using the GEPIA based on all cancer and normal tissue datasets compared in **Figure 1A**. **(A)** Protein-protein interaction network of top 100 RAD51 correlated genes. **(B)** GO Biological Processes enrichment analysis of top 100 RAD51 correlated genes. **(C)** KEGG Pathway enrichment analysis of top 100 RAD51 correlated genes. **(D)** Immunologic signatures enrichment analysis of top 100 RAD51 correlated genes. All the enrichment analyses and enrichment network constructions were conducted using the Metascape. **(E)** Immunofluorescence staining of the subcellular distribution of RAD51 within the nucleus, endoplasmic reticulum (ER), and microtubules of A431 squamous carcinoma cells, U-2 OS osteosarcoma cells, and GBM cells. **(F)** Plots of single-cell RNA-sequencing data from the FUCCI U-2 OS osteosarcoma cell line, showing the correlation between RAD51 mRNA expression and cell cycle progression. **(G)** The correlation of OCLR scores and RAD51 in TCGA cancer data. OCLR algorithm was used to calculate the mRNAsi (OCLR scores) for the evaluation of stemness. **(H)** The correlation of tumor mutational burden (TMB) and RAD51 in TCGA cancer data. **(I)** The correlation of microsatellite instability (MSI) and RAD51 in TCGA cancer data.

To predict the potential role RAD51 plays in cancers, RAD51 correlated expressing genes were identified, as shown in **S-Table 2**. A protein-protein interaction network was constructed using the STRING to display the potential association of RAD51 and these genes (**Figure 5A**). The top 100 correlated genes were further analyzed in the enrichment study. The GO biological processes enrichment results showed that RAD51 was mainly associated with terms related to the cell cycle, such as “DNA replication”, “DNA repair”, and “cytokinesis” (**Figure 5B**). The KEGG pathways enrichment showed that the “cell cycle” was the most enriched pathway (**Figure 5C**). In addition, this study also conducted an immunologic signature enrichment analysis using the GSEA GSEA Molecular Signatures Database. Results revealed that these genes were potentially associated with CD8 T-cells, CD4 T-cells, B cells, and cytokines, such as IL4 and IL6 (**Figure 5D**). The immunofluorescence staining of the subcellular distribution of RAD51 within the nucleus, endoplasmic reticulum (ER), and microtubules of A431 squamous carcinoma cells, U-2 OS osteosarcoma cells, and GBM cells showed that RAD51 is mostly expressed in nucleoli rim, which accounted for its association to the cell cycle (**Figure 5E**). The cell cycle of the U-2 OS osteosarcoma cell line was analyzed as a cancer cell example to observe the change of RAD51 expressed during the cell cycle. Results showed that RAD51 kept raising at the early stage of the cell cycle and started to recover after about 9 hours. Thus, the S-tr phase expressed the highest RAD51 (**Figure 5F**). The alteration of RAD51 during the cell cycle further indicated its critical role in regulating the cell cycle of cancer cells. Moreover, the correlations of RAD51 to cancer stemness, TMB, and MSI were also analyzed across different cancer types. Results showed that RAD51 was positively correlated with the stemness of 27 types of cancer. Among these cancer types, RAD51 was strongly correlated to the stemness in 8 cancer types, such as breast invasive carcinoma (BRCA), esophageal carcinoma (ESCA), lung cancer (LUAD&LUSC), and stomach adenocarcinoma (STAD), where the coefficients were over 0.5 (**Figure 5G**). In TMB and MSI analysis, most of the cancer types were not correlated to the RAD51 level, except for the TMB of 6 cancer types which were weakly correlated to the RAD51 level (**Figures 5H–I**).

### Associations of RAD51 and Immunomodulators in Cancers

Pan-cancer analyses aimed at depicting the immunological role of RAD51 are critical in determining the types of cancers that may benefit from anti-RAD51 immunotherapy or immunotherapy prediction by RAD51. Results of correlation analysis of RAD51 and immunomodulators revealed that RAD51 might be associated with multiple immunomodulators in different cancer types. Strikingly, RAD51was positively correlated with a majority of immunomodulators in thyroid carcinoma (THCA) and kidney renal clear cell carcinoma (KIRC). RAD51 was negatively correlated with a majority of Immunol inhibitors, Immunol stimulators, and Chemokine receptors in esophageal carcinoma (ESCA) and glioblastoma multiforme (GBM). RAD51 was negatively correlated with many MHC molecules in multiple cancer types, such as adrenocortical carcinoma (ACC) and lung cancer (LUAD&LUSC). RAD51 was also positively correlated with most MHC molecules in liver hepatocellular carcinoma (LIHC) and low-grade glioma (LGG). Therefore, this study suggested RAD51 might associate with the immune regulation in these cancer types. From the other dimension of the correlation, KDR, CCL14, CXCL12, IL6R, TNFSF13, and CX3CR1 were negatively correlated with RAD51 in multiple cancer types, while LAG3, CD276, MICB, ULBP1, TAP1, and TAP2 were positively correlated with RAD51 in most cancer types, indicating that these molecules can be potential immunotherapy targets of RAD51 across cancer types. Detailed results of the correlations were displayed in **S-Figure 2**.

### Associations of RAD51 and Immune Subtypes of Cancers

To investigate whether RAD51 can potentially affect immune subtypes (Immune Landscape) of cancers, this study also analyzed associations between RAD51 expression and immune subtypes across human cancers. Results showed that expression of RAD51 was significantly different across immune subtypes in 11 cancer types. In most of the cancer types, the C3 (inflammatory) immune subtype expressed lower RAD51 than the other immune subtypes (**S-Figure 3**). Thus, RAD51 might potentially be an exclusion biomarker for the inflammatory immune subtype in these cancer types.

### Associations of RAD51 and Immune Cell Infiltration Across Cancers

To further study the association of RAD51 and cancer immune microenvironments, immune cell infiltration levels were estimated using the XCELL algorithm. The correlation analysis of RAD51 and immune cell infiltration levels revealed that RAD51 might be closely associated with cancer immune microenvironments in some cancer types. RAD51 was negatively associated with stroma scores in 22 cancer types. LGG was the only cancer type whose stroma score was weakly and positively correlated to RAD51. For microenvironmental scores, RAD51 was negatively associated with the scores in 14 cancer types, while in 3 cancer types, RAD51 was positively associated with the microenvironmental scores. On the other hand, RAD51 was positively correlated to immune scores in 6 cancer types but negatively correlated to immune scores in 9 cancer types. In detail, generally, RAD51 was negatively associated with most of the XCELL scores except for T cell CD4+ (Th1 and TH2) and common lymphoid progenitor scores. where RAD51 was positively correlated to T cell CD4+ Th1, T cell CD4+ TH2, and common lymphoid progenitor scores in most of the cancer types, especially T cell CD4+ TH2 which was strongly correlated with RAD51 in most of the cancer types. In 5 cancer types, RAD51 was strongly and negatively associated with the majority of the XCELL scores, while in 3 cancer types, RAD51 was strongly and positively associated with the majority of the XCELL scores. Most strikingly, in thymoma (THYM), most of the immune cell scores were strongly and positively associated with RAD51, but most of the stroma cell scores were strongly and negatively associated with RAD51. Detailed results of the correlations were displayed in **S-Figure 4**.

### The RAD51 Expression of Immune Cells in Cancers

As most of the above analysis demonstrated that RAD51 level in cancer was associated with immune cells, especially T cells, to further understand the distribution of RAD51 in tissue samples, single-cell expression data were collected and explored to compare the RAD51 expression in different cell types (including cancer cells and non-cancer cells) across multiple cancer types. Interestingly, results revealed that proliferating T cells was the cell type that expressed the highest RAD51 across most of the cancer samples analyzed. Generally, although with limited malignant cells containing samples, results showed that malignant cells expressed relatively high RAD51 than the other cells but the expression was much lower than that in proliferating T cells. Therefore, this study proposed that the levels of RAD51 in these cancer types depended on proliferating T cells if there is a large proportion of this type of T cells. These results account for the previous suggestion that RAD51 was closely associated the T cell activities. Detailed results of the RAD51 expressions of cells were displayed in **S-Figure 5**.

### The Predictive Value of RAD51 for Immune Therapy of Cancers

**Figure 6:**
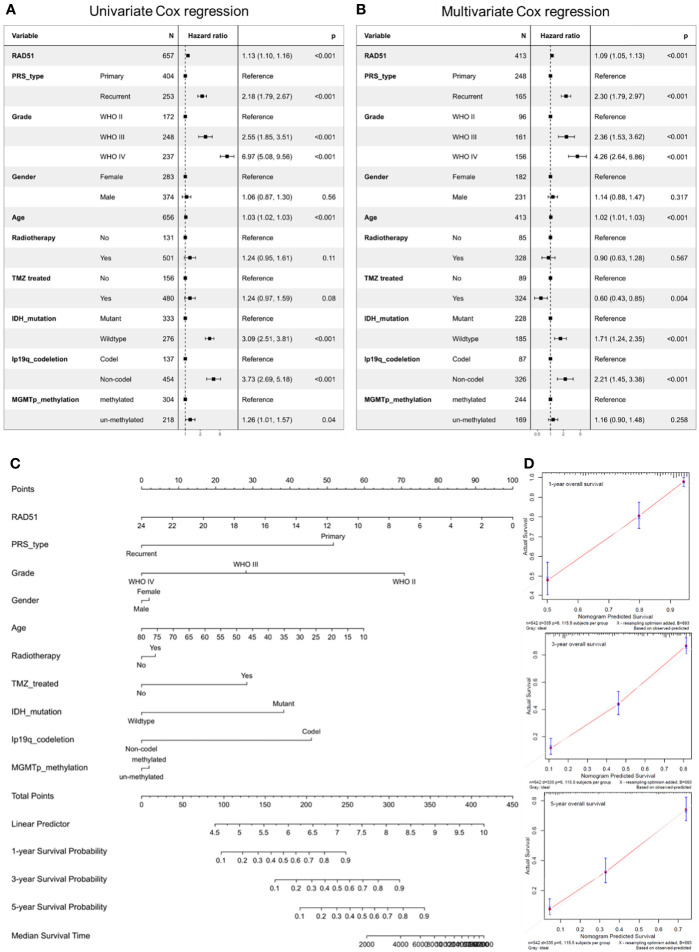
Construction of a prognostic model of RAD51 for overall glioma. The CGGA-mRNAseq_693 cohort was used to construct the model. **(A)** Univariate Cox regression analysis of overall survival of glioma patients. **(B)** Multivariate Cox regression analysis of overall survival of glioma patients. **(C)** Nomogram for the prediction of overall survival of glioma patients. **(D)** Calibration plots of the nomogram.

This study evaluated the biomarker relevance of RAD51 by comparing RAD51 expression with standardized biomarkers based on their predictive power of response outcomes and overall survival of ICB sub-cohorts. Results showed that RAD51 expression had AUCs of over 0.5 in 12 of the 23 ICB subcohorts. Data suggested that RAD51 exhibited an even higher predictive value than TMB, T.Clonality, and B. Clonality, which had AUC values of over 0.5 in only 7, 9, and 6 ICB sub-cohorts respectively. However, the predictive value of RAD51 was similar to the MSI score, which had AUCs over 0.5 in 12 ICB subcohorts, but the predictive value of RAD51 was lower than CD27A, TIDE, IFNG, and CD8 (**S-Figure 6A**). To further evaluate the value of RAD51 for clinical immune therapy, this study compared immune checkpoint blockade (ICB) responses of RAD51 low (0-50%) and high (50-100%) samples across different cancer types. Potential ICB response was predicted using the Tumor Immune Dysfunction and Exclusion (TIDE) algorithm. The calculation showed that the TIDE of 9 cancer types was significantly different between RAD51 high and low groups. For example, in thyroid carcinoma (THCA), 33 out of 128 (25.7%) patients in the RAD51 high group were predicted to respond to ICB treatment, while 42 out of 128 (32.8%) patients in the RAD51 low group were predicted to respond to ICB treatment. Thymoma (THYM), lung squamous cell carcinoma (LUSC), and breast invasive carcinoma (BRCA) had relatively large p-value, thus they were not as striking (**S-Figure 6B**). These results suggested that, in thyroid carcinoma (THCA), lung adenocarcinoma (LUAD), liver hepatocellular carcinoma (LIHC), overall glioma, and kidney renal clear cell carcinoma (KIRC), patients with a lower RAD51 expression were much more likely to respond to ICB immunotherapy. Therefore, this study proposed that RAD51 can be a predictive factor for ICB therapy in these cancer types.

### The Predictive Value of RAD51 for Drug Therapy of Cancers

Gene expressions influence clinical responses to drug treatment and these genes are potential biomarkers for drug screening. To investigate whether the RAD51 level was associated with drug sensitivity, the expression of RAD51 was performed by Spearman correlation analysis with the drug sensitivity (IC_50_) to multiple anti-cancer drugs from the GDSC and CTRP databases. Drug sensitivity and gene expression profiling data of cancer cell lines in GDSC and CTRP were integrated for investigation. Results showed that RAD51 expression was negatively correlated with sensitivities of most drugs. In GDSC, NPK76-II-72-1, PIK-93, and Vorinostat were the top three most negatively correlated drugs, while Trametinib was the only positively correlated drug (**S-Figure 7A**). In CTRP, the sensitivities of almost all drugs were negatively correlated with RAD5 (**S-Figure 7B**). These data suggested that RAD51 can be a biomarker for the prediction of drug therapy. In addition, this study also analyzed the association of RAD51 and chemotherapy by plotting the expression of RAD51 in responder and nonresponder, chemotherapy predictive ROC plot, and chemotherapy response in RAD51 quartiles expression groups in four cancer types. Results showed that in breast invasive carcinoma (BRCA) and colon adenocarcinoma (COAD), the chemotherapy responder group expressed lower RAD51 than the non-responder group respectively. However, in ovarian serous cystadenocarcinoma (OV) and glioblastoma multiforme (GBM), the chemotherapy responder group expressed higher RAD51 than the non-responder group (**S-Figure 7C**). These data indicated that RAD51 had potential chemotherapy predictive value but predictive patterns might vary from different cancer types.

### The Application of RAD51 in Overall Glioma Prognosis

To demonstrate the practicable clinical application of RAD51, this study focused on one type of cancer glioma, and develop strategies for the application of RAD51 in overall glioma prognosis. The above results indicated that RAD51 was significantly associated with the survival of LGG but not GBM. Glioma has been classified into four grades based on histology and clinical criteria: grade I, II, III, and IV (34). Grade I glioma, which usually occurs in children, is generally beneficial and can be cured by surgical resection. Grade I glioma has been believed to be different from grade II-IV glioma, which primarily occurs in adults. Grade II and III gliomas are often referred to as “low-grade glioma (LGG)” while grade IV glioma is referred to as “highest grade glioma”, “glioblastoma”, or “glioblastoma multiforme (GBM)” (35). In this context, this study found that not only overall glioma expressed significantly higher RAD51 than normal brain tissues, but also higher-grade gliomas expressed significantly higher RAD51 than lower-grade gliomas (**S-Figure 8A**). Sankey’s diagram of overall glioma data from TCGA (LGG+GBM) showed that RAD51 levels might associate with the survival of patients (**S-Figure 8B**). The KM plotting of high and low RAD51 patients of overall glioma showed that RAD51 was significantly associated with overall survival in TCGA (LGG+GBM) cohort (n=332) (**S-Figure 8C**). To validated this conclusion, three independent external glioma cohorts were analyzed, including CGGA_mRNAseq_693 primary (n=404), CGGA_mRNAseq_325 primary (n=222), and CGGA_mRNA-array_301 primary (n=262). Results showed that in all three of these validation cohorts, the RAD51 high group had a significantly lower survival rate compared to the RAD51 low group respectively. Therefore, the survival association of RAD51 in overall glioma was confirmed (**S-Figures 8D–F**).

### Construction of RAD51 Survival Prediction Model for Glioma

To screen risk factors for the clinical survival prediction model for glioma, this study conducted a univariate Cox analysis of overall survival and clinical variables including RAD51 expression, PRS type, Grade, gender, age, radiotherapy, Temozolomide (TMZ) treatment, IDH mutation, 1p19q codeletion, and MGMTp methylation. Results revealed that RAD51 expression, PRS type, Grade, Age, IDH mutation, 1p19q codeletion, and MGMTp methylation were significantly associated with the overall survival of glioma patients (**Figure 6A**). To rule out the potential inter-association of these factors, a multivariate Cox regression analysis for these variables was conducted. Results showed that RAD51 expression, PRS type, Grade, Age, TMZ treatment, IDH mutation, and 1p19q codeletion were significantly and independently provided prediction confidence for overall survival of glioma (**Figure 6B**). Based on multivariate Cox analysis, a nomogram was constructed for the prediction of 1-, 3-, 5- year survival of overall glioma patients (**Figure 6C**). The prediction results of the nomogram calibration curves of 1-, 3-, 5- year overall survival was generally consistent with all patients’ observation results (**Figure 6D**).

## Discussion

In this study, the bioinformatic data supported the potential values of RAD51 for clinical cancer management, regarding cancer diagnosis, prognosis, and therapeutic prediction. Most of the similar studies exploring cancer biomarkers focused on one cancer type (36, 37), while this study screened for 33 cancer types. Pan-cancer analysis has been reported previously (38–40) as a bioinformatic methodology to screen cancer types that are interesting to be further explored. One strength of the study is the use of TCGA and the analysis of different cancers, enabling us to have an overview of the biomarker value of RAD51 in cancers.

Although the function of RAD51 in cell growth has been well defined (41), the association of RAD51 and human diseases has not been studied wildly and the clinical use of RAD51 as a biomarker for cancer has not been developed. Only a few studies reported the potential association of RAD51 and human diseases. The mutation of RAD51 was thought to be associated with some dysfunction in the human body. For example, RAD51 mutation has been found to be associated with the disorder in congenital mirror movements (42) and Fanconi anemia (43, 44). For cancers, only breast cancer was suggested to be associated with the mutation of RAD51 (45, 46). The RAD51 genetic analysis of this study suggested that RAD51 genetic alterations might not be the major factor that drives the development of cancers, because the genetic alteration rate of RAD51 in cancers was relatively low. For most cancer cases, RAD51 did not have genetic alterations but had abnormal expression levels. Considering that the purpose of this study is to provide evidence for clinical applications, topics that have a wider range of applications would be more interested in this study. Therefore, this study focused mostly on the expression level of RAD51 rather than the genetic alteration of RAD51.

On one hand, the present study demonstrated that RAD51 has good diagnostic power for multiple cancer types because RAD51 was overexpressed in these cancer types compared to normal tissues. RAD51, as a critical protein that regulated DNA repair, was supposed to be overexpressed in proliferating cells. Cancer cells adapt cellular metabolism to cope with their high proliferation rate (47), hence, it is reasonable that cancer cells have a higher expression of RAD51 than normal cells. Data of this study showed that RAD51 is mostly expressed in nucleoli rim and the cell cycle analysis in this study showed that, at the early phase of the cell cycle, RAD51 increased dramatically. This is the phase when most DNA repairments happen (48). The enrichment results suggested that most of the co-expressed genes of RAD51 were associated with the cell cycle. Based on the understanding of the known function of RAD51, RAD51 is not likely to be an upstream driver for the cell cycle, but a downstream gene that is activated by the cell cycle activities and the requirement of cells for DNA repairments. In this context, new approaches to pharmacologically inhibit RAD51 can not inhibit the cells proliferation of cancer cells. However, the upregulation of the RAD51 indicates that more DNA error occurs in the cells that recruit more DNA repairers. Since genetic instability is one of the hallmarks of cancer, the DNA repairer, RAD51, as a biomarker for the distinguishment of cancerness of a cell, appears to be particularly reliable. Results showed that RAD51 was significantly overexpressed in 28 types of cancer. Acute myeloid leukemia (LAML) is the only one expressed lower RAD51 in cancer than in normal tissues. These data suggested that RAD51 have a common cancer-noncancer expression pattern for most of the cancer types and can potentially be a diagnostic biomarker for most of these cancer types.

On the other, RAD51 can be used as a prognostic biomarker for certain cancer types as the expression of RAD51 was found associated with patients’ survival. The study of RAD51 as biomarkers is a rather popular topic in the field. The prognostic value of RAD51 has been reported in colon cancer (15), pancreatic cancer (16), breast cancer (19), and liver cancer (18). Here, this study additionally reported the survival association of RAD51 in 8 cancer types, enlarging the cancer type range that RAD51 might be applied in prognosis. Different from most of the previous pan-cancer studies, to further expand the clinical significance of this paper, a prognostic model of RAD51 for glioma was constructed as an example application of RAD51 for these cancer types. The prognostic power of RAD51 in glioma has not been reported, as in most of the studies, high- and low-grade glioma were studied separately. However, strictly, GBM (high-grade glioma) and LGG (low-grade glioma) are all gliomas. LGG is Grade II and III gliomas, while GBM is grade IV glioma. This study had shown that RAD51 had strong prognostic power for overall glioma. The nomogram demonstrated the practical and practicality and feasibility of the use of RAD51 for overall glioma prognosis.

One of the deeply explored fields of this study is the association of RAD51 and cancer immunity. An interesting finding was that RAD51 was distinguished and highly expressed in T cells in the cancer samples. This might confuse us that whether the level of RAD51 was only dependent on the T cells infiltration levels. This study proposed that the levels of RAD51 in some cancer types depended on proliferating T cells if there is a large proportion of T cells, but for most samples, T cells have a relatively low proportion and the major expression of RAD51 was from cancer cells. Nevertheless, the striking finding of these results was the association of T cells and RAD51 in cancers, which was consistent with previous studies (49–51).

Another inference of the RAD51 T cell association is that RAD51 might potentially affect immune therapy. As one of the major cells in the immune defense system against cancer, T cells play essential roles in cancer immunity. T cells move through tissues, scanning for MHC-peptide complexes that specifically activate their T cell receptors and can also sense a variety of signals that can alert them to cancer (52). Therefore, T cells are an important part of immune therapy. Results in this study suggested that RAD51 can be a predictive factor for the response to immune therapy in multiple cancer types. RAD51 was closely correlated with immune cell infiltrations and immune-molecule expressions. In the past few years, immunotherapy has been developed for cancer treatment and has achieved significant improvements in cancer therapies (53). Immunotherapy treats cancer *via* immune checkpoint blockade (ICB). For example, the programmed cell death protein 1 (PD-1) blockade has been found to be effective in the treatment of multiple cancer types (54–57). However, the effectiveness of ICB treatment may vary from patient to patient (57). Thus, one of the most critical challenges for ICB treatment that need to be overcome is the identification of discrepancies between different genomic subtypes in their response to ICB treatment. In this study, the analysis demonstrated the potential use of RAD51 as a sign of the multiple cancer types immune microenvironment and RAD51 can be a potential predictive biomarker for the response to immune therapy. In addition, targeting RAD51 was reported to enhance chemosensitivity of adult T-cell leukemia-lymphoma cells by reducing DNA double-strand break repair. This study demonstrated that RAD51 might potentially associate with the drug sensitivities of multiple anti-cancer drugs. Many of the analyzed drugs can inhibit the proliferation or impede the cell cycle of cancer cells. As mentioned above, RAD51 might not be the upstream target but a reliable biomarker to predict the sensitivity of the drugs. However, these results require further validation in the future.

## Conclusion

RAD51 is a clinical valuable biomarker for multiple cancer types, regarding its potential power for diagnosis, prognosis, and therapeutic prediction.

## Data Availability Statement

The original contributions presented in the study are included in the article/**Supplementary Material**. Further inquiries can be directed to the corresponding authors.

## Author Contributions

All the works were conducted by HL. JW supervised the project and reversed the manuscript. All authors contributed to the article and approved the submitted version.

## Funding

This study received funding from Biocomma Limited. The funder was not involved in the study design, collection, analysis, interpretation of data, the writing of this article or the decision to submit it for publication.

## Conflict of Interest

Author HL was employed by Biocomma Limited. 

The remaining author declare that the research was conducted in the absence of any commercial or financial relationships that could be construed as a potential conflict of interest.

## Publisher’s Note

All claims expressed in this article are solely those of the authors and do not necessarily represent those of their affiliated organizations, or those of the publisher, the editors and the reviewers. Any product that may be evaluated in this article, or claim that may be made by its manufacturer, is not guaranteed or endorsed by the publisher.

## References

[1] Siegel RL Miller KD Fuchs HE Jemal A. Cancer Statistics, 2021. CA Cancer J Clin (2021) 71:7–33. doi: 10.3322/caac.21654 33433946

[2] Tomczak K Czerwińska P Wiznerowicz M. The Cancer Genome Atlas (TCGA): An Immeasurable Source of Knowledge. Contemp Oncol (Pozn) (2015) 19:A68–77. doi: 10.5114/wo.2014.47136 PMC4322527 25691825

[3] Lonsdale J Thomas J Salvatore M Li G Phillips R Lo E Shad S The Genotype-Tissue Expression (GTEx) Project. Nat Genet (2013) 45:580–5. doi: 10.1038/ng.2653 PMC4010069 23715323

[4] Lonsdale J Thomas J Salvatore M Phillips R Lo E Shad S. Chinese Glioma Genome Atlas (CGGA): A Comprehensive Resource With Functional Genomic Data From Chinese Glioma Patients. Genomics Proteomics Bioinf (2021) 19:1–12. doi: 10.1016/j.gpb.2020.10.005 PMC8498921 33662628

[5] Hanahan D Weinberg RA. Hallmarks of Cancer: The Next Generation. Cell (2011) 144:646–74. doi: 10.1016/j.cell.2011.02.013 21376230

[6] Piazza A Heyer WD. Multi-Invasion-Induced Rearrangements as a Pathway for Physiological and Pathological Recombination. Bioessays (2018) 40:e1700249. doi: 10.1002/bies.201700249 29578583 PMC6072258

[7] Ranjha L Howard SM Cejka P. Main Steps in DNA Double-Strand Break Repair: An Introduction to Homologous Recombination and Related Processes. Chromosoma (2018) 127:187–214. doi: 10.1007/s00412-017-0658-1 29327130

[8] Bell JC Kowalczykowski SC. Mechanics and Single-Molecule Interrogation of DNA Recombination. Annu Rev Biochem (2016) 85:193–226. doi: 10.1146/annurev-biochem-060614-034352 27088880

[9] Naipal KA Verkaik NS Ameziane N van Deurzen CH Ter Brugge P Meijers M. Functional Ex Vivo Assay to Select Homologous Recombination-Deficient Breast Tumors for PARP Inhibitor Treatment. Clin Cancer Res (2014) 20:4816–26. doi: 10.1158/1078-0432.CCR-14-0571 24963051

[10] Qiao GB Wu YL Yang XN Zhong WZ Xie D Guan XY. High-Level Expression of Rad51 is an Independent Prognostic Marker of Survival in Non-Small-Cell Lung Cancer Patients. Br J Cancer (2005) 93:137–43. doi: 10.1038/sj.bjc.6602665 PMC2361489 15956972

[11] Vispé S Cazaux C Lesca C Defais M. Overexpression of Rad51 Protein Stimulates Homologous Recombination and Increases Resistance of Mammalian Cells to Ionizing Radiation. Nucleic Acids Res (1998) 26:2859–64. doi: 10.1093/nar/26.12.2859 PMC147643 9611228

[12] Slupianek A Hoser G Majsterek I Bronisz A Malecki M Blasiak J. Fusion Tyrosine Kinases Induce Drug Resistance by Stimulation of Homology-Dependent Recombination Repair, Prolongation of G(2)/M Phase, and Protection From Apoptosis. Mol Cell Biol (2002) 22:4189–201. doi: 10.1128/MCB.22.12.4189-4201.2002 PMC133854 12024032

[13] Hansen LT Lundin C Spang-Thomsen M Petersen LN Helleday T. The Role of RAD51 in Etoposide (VP16) Resistance in Small Cell Lung Cancer. Int J Cancer (2003) 105:472–9. doi: 10.1002/ijc.11106 12712436

[14] Liu Y Burness ML Martin-Trevino R Guy J Bai S Harouaka R. RAD51 Mediates Resistance of Cancer Stem Cells to PARP Inhibition in Triple-Negative Breast Cancer. Clin Cancer Res (2017) 23:514–22. doi: 10.1158/1078-0432.CCR-15-1348 28034904

[15] Lee JH Bae AN Jung AS. Clinicopathological and Prognostic Characteristics of RAD51 in Colorectal Cancer. Med (Kaunas) (2020) 56(2):48. doi: 10.3390/medicina56020048 PMC7073956 31973027

[16] Nagathihalli NS Nagaraju G. RAD51 as a Potential Biomarker and Therapeutic Target for Pancreatic Cancer. Biochim Biophys Acta (2011) 1816:209–18. doi: 10.1016/j.bbcan.2011.07.004 21807066

[17] Bhattacharya S Srinivasan K Abdisalaam S Su F Raj P Dozmorov I. RAD51 Interconnects Between DNA Replication, DNA Repair and Immunity. Nucleic Acids Res (2017) 45:4590–605. doi: 10.1093/nar/gkx126 PMC5416901 28334891

[18] Xu H Xiong C Chen Y Zhang C Bai D. Identification of Rad51 as a Prognostic Biomarker Correlated With Immune Infiltration in Hepatocellular Carcinoma. Bioengineered (2021) 12:2664–75. doi: 10.1080/21655979.2021.1938470 PMC8806544 34115569

[19] Li F Zhang Y Shi Y Liu S. Comprehensive Analysis of Prognostic and Immune Infiltrates for RAD51 in Human Breast Cancer. Crit Rev Euk Gene Expr (2021) 31:71–9. doi: 10.1615/CritRevEukaryotGeneExpr.2021038876 34587437

[20] Cerami E Gao J Dogrusoz U Gross BE Sumer SO Aksoy BA. The Cbio Cancer Genomics Portal: An Open Platform for Exploring Multidimensional Cancer Genomics Data. Cancer Discov (2012) 2:401–4. doi: 10.1158/2159-8290.CD-12-0095 PMC3956037 22588877

[21] Koscielny G An P Carvalho-Silva D Cham JA Fumis L Gasparyan R. Et Al: Open Targets: A Platform for Therapeutic Target Identification and Validation. Nucleic Acids Res (2017) 45:D985–d994. doi: 10.1093/nar/gkw1055 27899665 PMC5210543

[22] Brouwer I Moschetti T Candelli A Garcin EB Modesti M Pellegrini L. Two Distinct Conformational States Define the Interaction of Human RAD51-ATP With Single-Stranded DNA. EMBO J (2018) 37. doi: 10.15252/embj.201798162 PMC5881629 29507080

[23] Rhodes DR Yu J Shanker K Deshpande N Varambally R Ghosh D. ONCOMINE: A Cancer Microarray Database and Integrated Data-Mining Platform. Neoplasia (2004) 6:1–6. doi: 10.1016/S1476-5586(04)80047-2 15068665 PMC1635162

[24] Tang Z Li C Kang B Gao G Li C Zhang Z. GEPIA: A Web Server for Cancer and Normal Gene Expression Profiling and Interactive Analyses. Nucleic Acids Res (2017) 45:W98–w102. doi: 10.1093/nar/gkx247 28407145 PMC5570223

[25] Szklarczyk D Gable AL Nastou KC Lyon D Kirsch R Pyysalo S. The STRING Database in 2021: Customizable Protein-Protein Networks, and Functional Characterization of User-Uploaded Gene/Measurement Sets. Nucleic Acids Res (2021) 49:D605–d612. doi: 10.1093/nar/gkaa1074 33237311 PMC7779004

[26] Zhou Y Zhou B Pache L Chang M Khodabakhshi AH Tanaseichuk O. Metascape Provides a Biologist-Oriented Resource for the Analysis of Systems-Level Datasets. Nat Commun (2019) 10:1523. doi: 10.1038/s41467-019-09234-6 30944313 PMC6447622

[27] Pontén F Jirström K Uhlen M. The Human Protein Atlas—a Tool for Pathology. J Pathol: A J Pathol Soc Great Britain Ireland (2008) 216:387–93. doi: 10.1002/path.2440 18853439

[28] Ru B Wong CN Tong Y Zhong JY Zhong SSW Wu WC. Et Al: TISIDB: An Integrated Repository Portal for Tumor–Immune System Interactions. Bioinformatics (2019) 35:4200–2. doi: 10.1093/bioinformatics/btz210 30903160

[29] Aran D Hu Z Butte AJ. Xcell: Digitally Portraying the Tissue Cellular Heterogeneity Landscape. Genome Biol (2017) 18:220. doi: 10.1186/s13059-017-1349-1 29141660 PMC5688663

[30] Sun D Wang J Han Y Dong X Ge J Zheng R. Et Al: TISCH: A Comprehensive Web Resource Enabling Interactive Single-Cell Transcriptome Visualization of Tumor Microenvironment. Nucleic Acids Res (2021) 49:D1420–d1430. doi: 10.1093/nar/gkaa1020 33179754 PMC7778907

[31] Fu J Li K Zhang W Wan C Zhang J Jiang P. Large-Scale Public Data Reuse to Model Immunotherapy Response and Resistance. Genome Med (2020) 12:21. doi: 10.1186/s13073-020-0721-z 32102694 PMC7045518

[32] Liu CJ Hu FF Xia MX Han L Zhang Q Guo AY. GSCALite: A Web Server for Gene Set Cancer Analysis. Bioinformatics (2018) 34:3771–2. doi: 10.1093/bioinformatics/bty411 29790900

[33] Fekete JT Győrffy B. ROCplot.org: Validating Predictive Biomarkers of Chemotherapy/Hormonal Therapy/Anti-HER2 Therapy Using Transcriptomic Data of 3,104 Breast Cancer Patients. Int J Cancer (2019) 145:3140–51. doi: 10.1002/ijc.32369 31020993

[34] Louis DN Ohgaki H Wiestler OD Cavenee WK Burger PC Jouvet A. The 2007 WHO Classification of Tumours of the Central Nervous System. Acta Neuropathol (2007) 114:97–109. doi: 10.1007/s00401-007-0243-4 17618441 PMC1929165

[35] Claus EB Walsh KM Wiencke JK Molinaro AM Wiemels JL Schildkraut JM. Survival and Low-Grade Glioma: The Emergence of Genetic Information. Neurosurg Focus (2015) 38:E6–6. doi: 10.3171/2014.10.FOCUS12367 PMC4361022 25552286

[36] Li Y Liu H. Clinical Powers of Aminoacyl tRNA Synthetase Complex Interacting Multifunctional Protein 1 (AIMP1) for Head-Neck Squamous Cell Carcinoma. Cancer biomark (2022). doi: 10.3233/CBM-210340 PMC12364190 35068446

[37] Li Y Liu H Han Y. Potential Roles of Cornichon Family AMPA Receptor Auxiliary Protein 4 (CNIH4) in Head and Neck Squamous Cell Carcinoma. researchsquare.com (2021) 2021. doi: 10.21203/rs.3.rs-845967/v1 PMC12364253 36404537

[38] Lu M Zhao B Liu M Wu L Li Y Zhai Y. Pan-Cancer Analysis of SETD2 Mutation and Its Association With the Efficacy of Immunotherapy. NPJ Precis Oncol (2021) 5:51. doi: 10.1038/s41698-021-00193-0 34127768 PMC8203790

[39] Luo Z Liu W Sun P Wang F Feng X. Pan-Cancer Analyses Reveal Regulation and Clinical Outcome Association of the Shelterin Complex in Cancer. Brief Bioinform (2021) 22(5):bbaa441. doi: 10.1093/bib/bbaa441 33497432

[40] Zhang J Jiang H Du K Xie T Wang B Chen C. Pan-Cancer Analyses Reveal Genomics and Clinical Characteristics of the Melatonergic Regulators in Cancer. J Pineal Res (2021) 71:e12758. doi: 10.1111/jpi.12758 34289167

[41] Bonilla B Hengel SR Grundy MK Bernstein KA. RAD51 Gene Family Structure and Function. Annu Rev Genet (2020) 54:25–46. doi: 10.1146/annurev-genet-021920-092410 32663049 PMC7703940

[42] Méneret A Trouillard O Dunoyer M Depienne C Roze E. “Congenital Mirror Movements”. In: Adam MP Ardinger HH Pagon RA Wallace SE Bean LJH Mirzaa G Amemiya A, editors. GeneReviews(®). Seattle (WA: University of Washington, Seattle (1993-2021). 25763452

[43] Wang AT Kim T Wagner JE Conti BA Lach FP Huang AL. A Dominant Mutation in Human RAD51 Reveals Its Function in DNA Interstrand Crosslink Repair Independent of Homologous Recombination. Mol Cell (2015) 59:478–90. doi: 10.1016/j.molcel.2015.07.009 PMC4529964 26253028

[44] Ameziane N May P Haitjema A van de Vrugt HJ van Rossum-Fikkert SE Ristic D. A Novel Fanconi Anaemia Subtype Associated With a Dominant-Negative Mutation in RAD51. Nat Commun (2015) 6:8829. doi: 10.1038/ncomms9829 26681308 PMC4703882

[45] Kato M Yano K Matsuo F Saito H Katagiri T Kurumizaka H. Identification of Rad51 Alteration in Patients With Bilateral Breast Cancer. J Hum Genet (2000) 45:133–7. doi: 10.1007/s100380050199 10807537

[46] Chen J Morrical MD Donigan KA Weidhaas JB Sweasy JB Averill AM. Tumor-Associated Mutations in a Conserved Structural Motif Alter Physical and Biochemical Properties of Human RAD51 Recombinase. Nucleic Acids Res (2015) 43:1098–111. doi: 10.1093/nar/gku1337 PMC4333388 25539919

[47] Sancho P Barneda D Heeschen C. Hallmarks of Cancer Stem Cell Metabolism. Br J Cancer (2016) 114:1305–12. doi: 10.1038/bjc.2016.152 PMC4984474 27219018

[48] Sancar A Lindsey-Boltz LA Unsal-Kaçmaz K Linn S. Molecular Mechanisms of Mammalian DNA Repair and the DNA Damage Checkpoints. Annu Rev Biochem (2004) 73:39–85. doi: 10.1146/annurev.biochem.73.011303.073723 15189136

[49] Yang M Tian X Fan Z Yu W Li Z Zhou J. Targeting RAD51 Enhances Chemosensitivity of Adult T−cell Leukemia−Lymphoma Cells by Reducing DNA Double−Strand Break Repair. Oncol Rep (2019) 42:2426–34. doi: 10.3892/or.2019.7384 PMC6859462 31638261

[50] Ramezani S Shirdel A Rafatpanah H Akbarin MM Tarokhian H Rahimi H. Assessment of HTLV-1 Proviral Load, LAT, BIM, C-FOS and RAD51 Gene Expression in Adult T Cell Leukemia/Lymphoma. Med Microbiol Immunol (2017) 206:327–35. doi: 10.1007/s00430-017-0506-1 28466382

[51] Flygare J Benson F Hellgren D. Expression of the Human RAD51 Gene During the Cell Cycle in Primary Human Peripheral Blood Lymphocytes. Biochim Biophys Acta (1996) 1312:231–6. doi: 10.1016/0167-4889(96)00040-7 8703992

[52] Restifo NP Dudley ME Rosenberg SA. Adoptive Immunotherapy for Cancer: Harnessing the T Cell Response. Nat Rev Immunol (2012) 12:269–81. doi: 10.1038/nri3191 PMC6292222 22437939

[53] Baxevanis CN Perez SA Papamichail M. Cancer Immunotherapy. Crit Rev Clin Lab Sci (2009) 46:167–89. doi: 10.1080/10408360902937809 19650714

[54] Xia L Liu Y Wang Y. PD-1/PD-L1 Blockade Therapy in Advanced Non-Small-Cell Lung Cancer: Current Status and Future Directions. Oncologist (2019) 24:S31–s41. doi: 10.1634/theoncologist.2019-IO-S1-s05 30819829 PMC6394772

[55] Mahoney KM Freeman GJ McDermott DF. The Next Immune-Checkpoint Inhibitors: PD-1/PD-L1 Blockade in Melanoma. Clin Ther (2015) 37:764–82. doi: 10.1016/j.clinthera.2015.02.018 PMC4497957 25823918

[56] Wang Y Li G. PD-1/PD-L1 Blockade in Cervical Cancer: Current Studies and Perspectives. Front Med (2019) 13:438–50. doi: 10.1007/s11684-018-0674-4 30826965

[57] Wang X Guo G Guan H Yu Y Lu J Yu J. Challenges and Potential of PD-1/PD-L1 Checkpoint Blockade Immunotherapy for Glioblastoma. J Exp Clin Cancer Res (2019) 38:87. doi: 10.1186/s13046-019-1085-3 30777100 PMC6380009

