# Supplementary material for: A Pan-Cancer Bioinformatic Analysis of RAD51 Regarding the Values for Diagnosis, Prognosis, and Therapeutic Prediction
Source: Front Oncol. 2022 Mar 10;12:858756. doi: 10.3389/fonc.2022.858756 (PMC8960930; doi:10.3389/fonc.2022.858756)
Supplement: Supplementary file 1 [file DataSheet_1.docx]

**Supplementary materials for:**

**A comprehensive pan-cancer analysis of RAD51**

Hengrui Liu

Biocomma Limited, Shenzhen, China

Correspondence: CommaBio, 1st Floor, Building 12, Zhonghaixin Innovation Industry City, No. 12, Ganli 6th Road, Gankeng Community, Jihua Street, Longgang District, Shenzhen, R. P. China, hengrui.liu@biocomma.cn

**Graphical abstract**

**
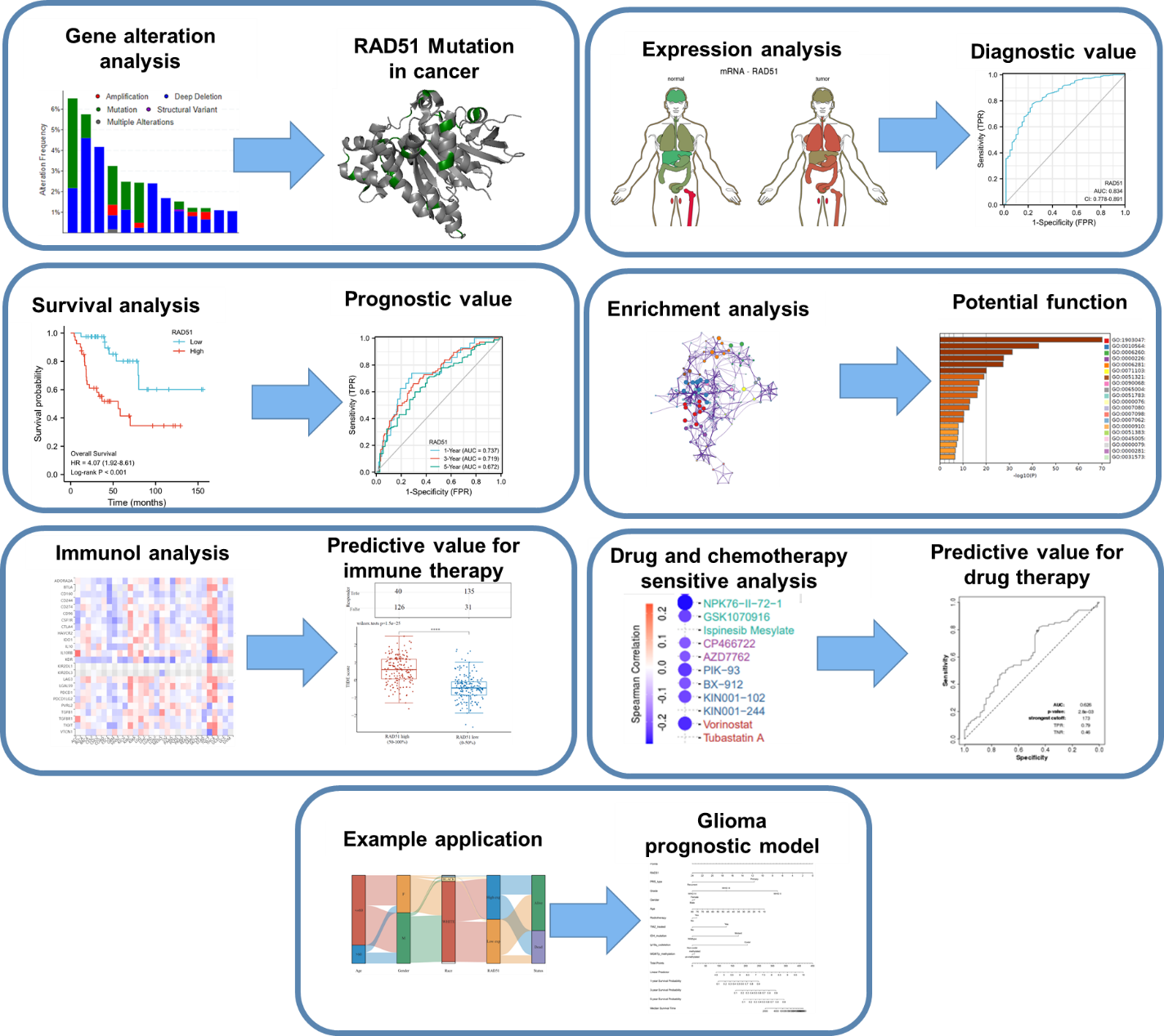
**


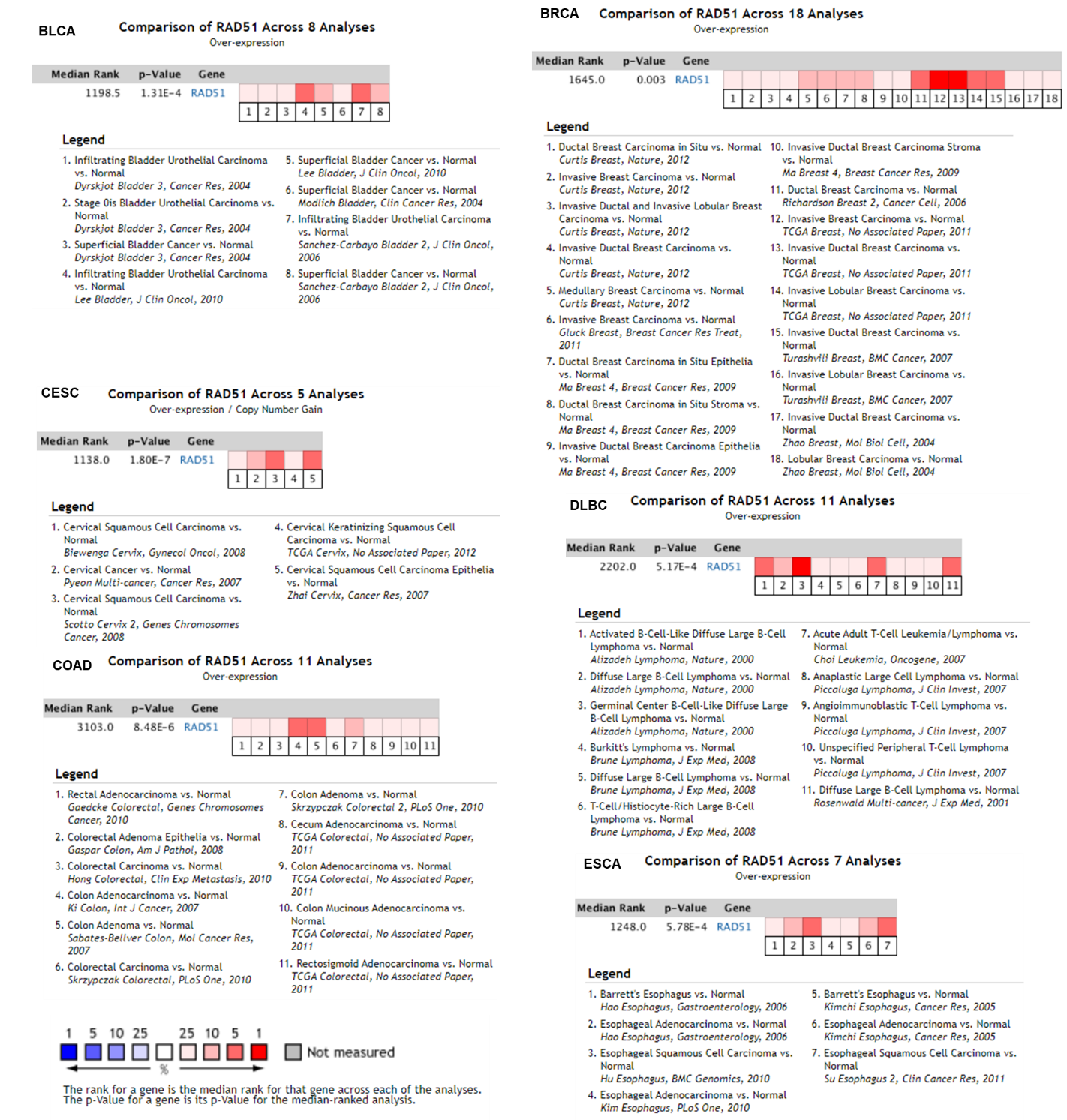


**S-Figure 1-1.** The overexpression or DNA copy number gain of RAD51 in different cancers versus normal tissues across multiple analyses (part 1). Multiple data sets were accessed and analyzed using the Oncomine. The heatmap showed the expression or copy number difference analysis between cancer and normal tissues. The legend listed the references for the data in the heatmap.


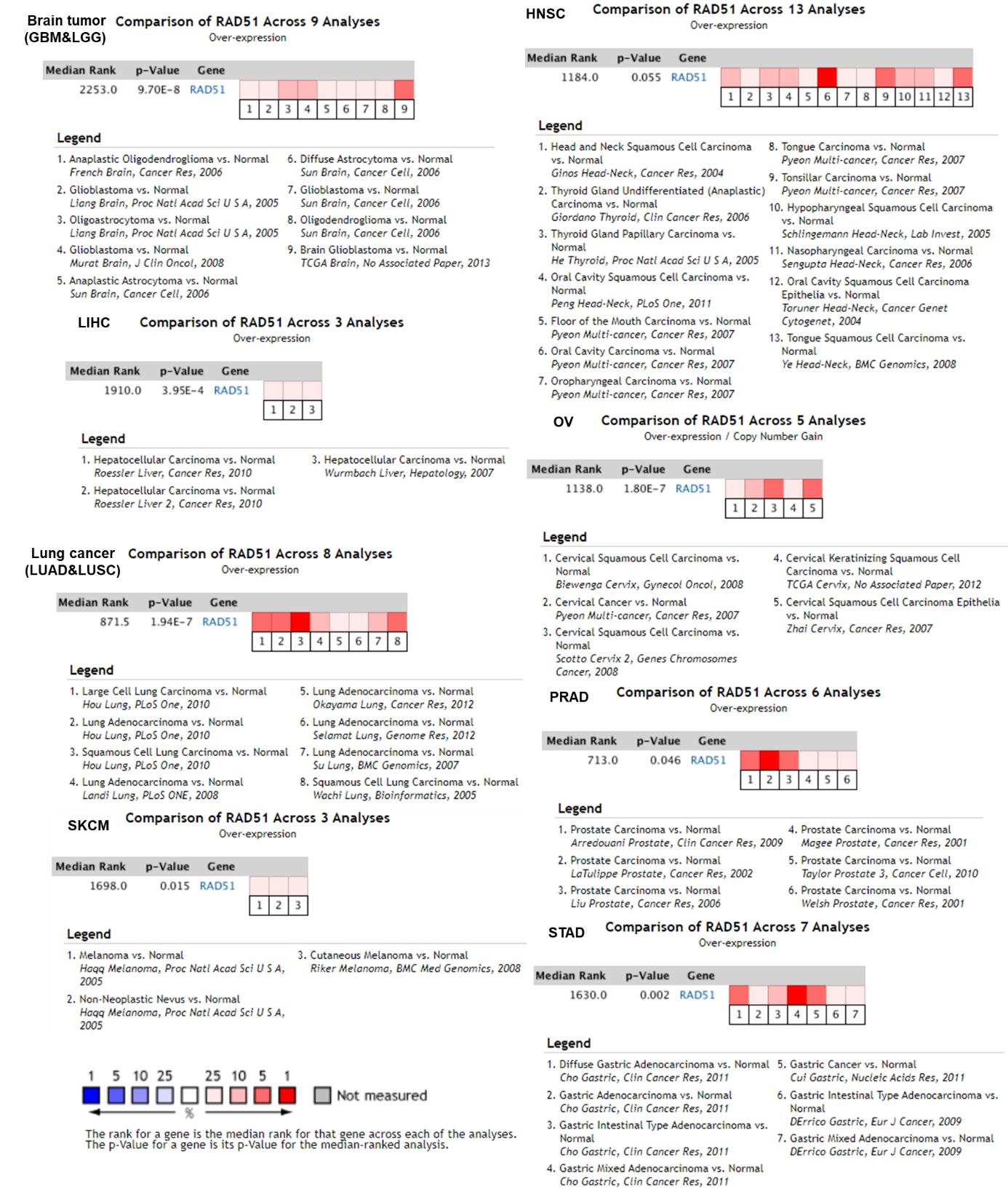


**S-Figure 1-2.** The overexpression or DNA copy number gain of RAD51 in different cancers versus normal tissues across multiple analyses (part 2). Multiple data sets were accessed and analyzed using the Oncomine. The heatmap showed the expression or copy number difference analysis between cancer and normal tissues. The legend listed the references for the data in the heatmap.


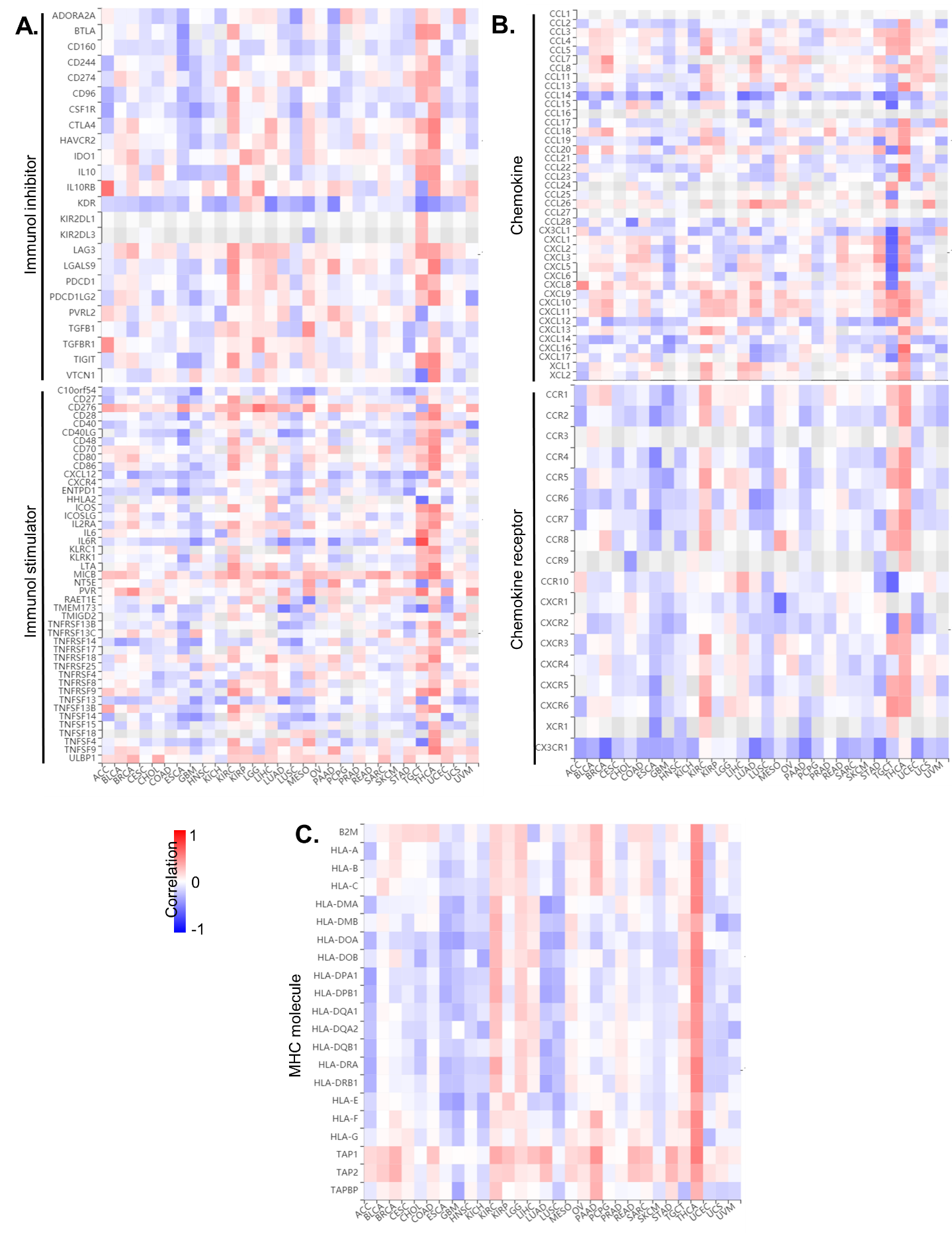


**S-Figure 2.** Immunomodulators association of RAD51 across cancer types. TCGA data were analyzed using the TISIDB. **A.** The correlation of RAD51 and Immunol inhibitors/stimulators. **B.** The correlation of RAD51 and Chemokines/Chemokine receptors. **C.** The correlation of RAD51 and MHC molecules.


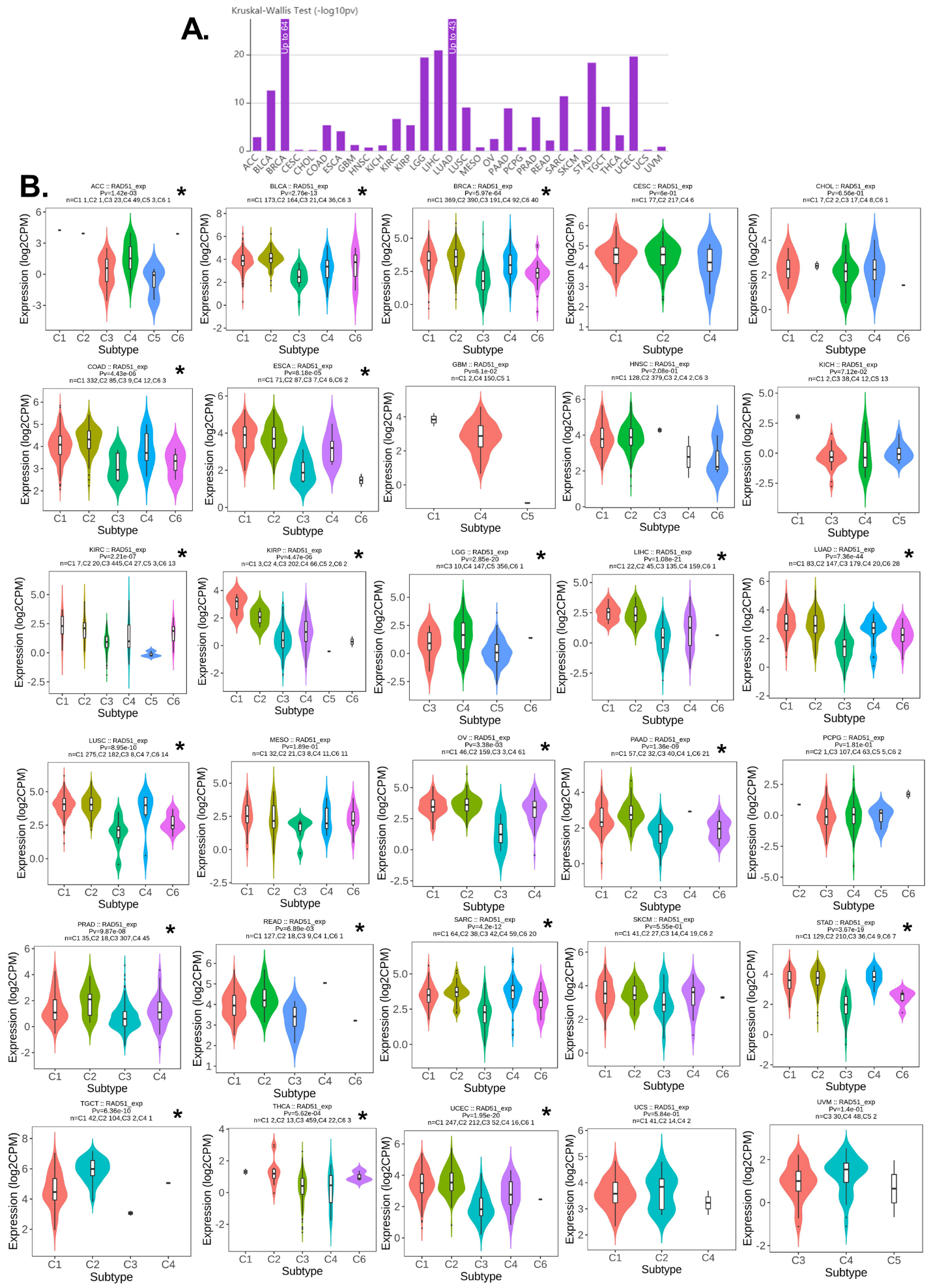


**S-Figure 3.** Associations between RAD51 expression and immune subtypes across human cancers. TCGA data were analyzed using the TISIDB. **A.** p-value of immune subtype difference in RAD51 expression across 30 cancer types. **B.** Detailed plottings of RAD51 expression in and immune subtypes across 30 cancer types. C1 (wound healing); C2 (IFN-gamma dominant); C3 (inflammatory); C4 (lymphocyte depleted); C5 (immunologically quiet); C6 (TGF-b dominant). (*P<0.05)


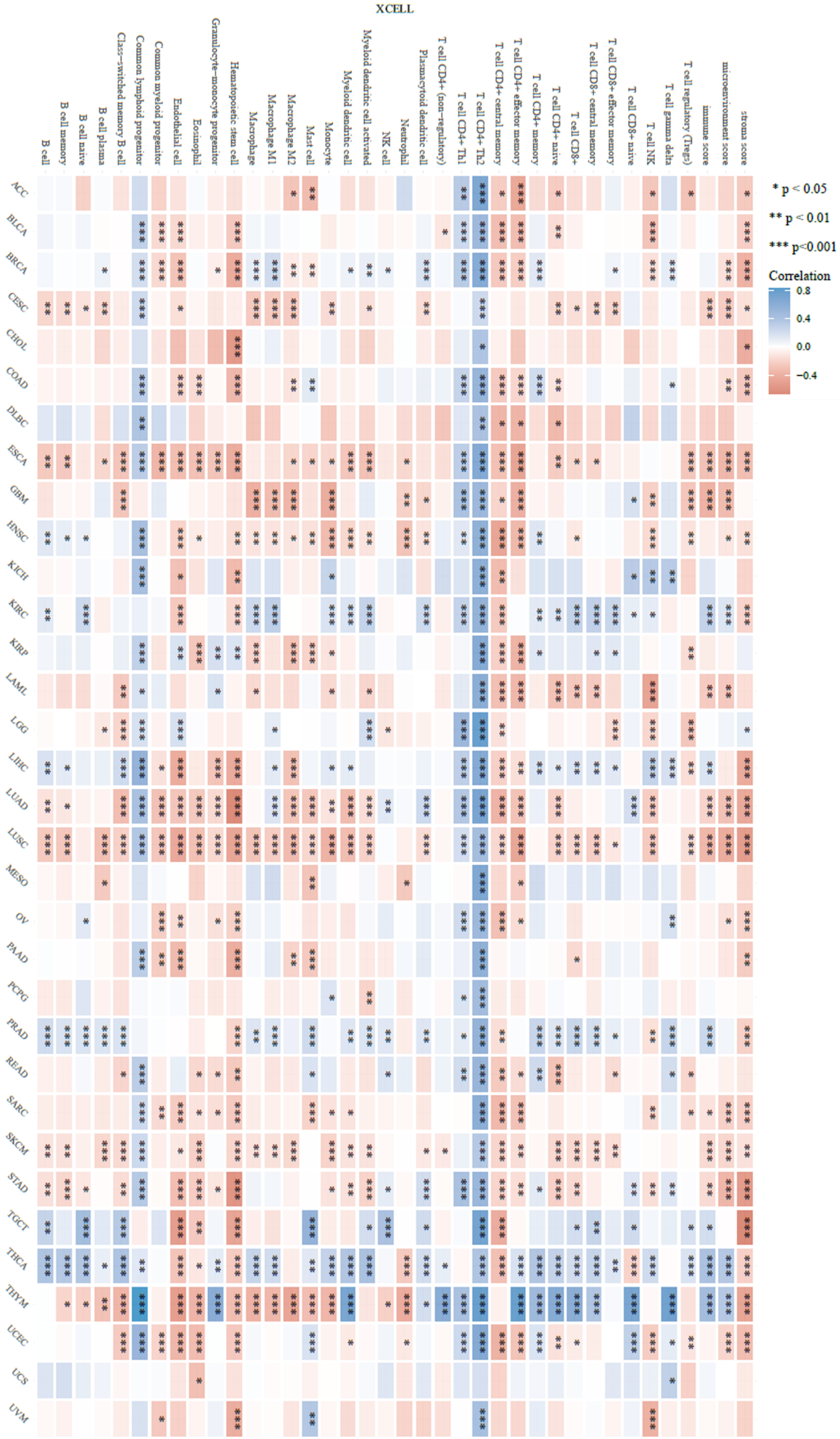


**S-Figure 4.** The correlation of RAD51 expression and immune cell infiltration levels. TCGA data were analyzed. The XCELL algorithms were used to estimate the immune cell infiltration levels. (Blue: positive correlation; red: negative correlation)


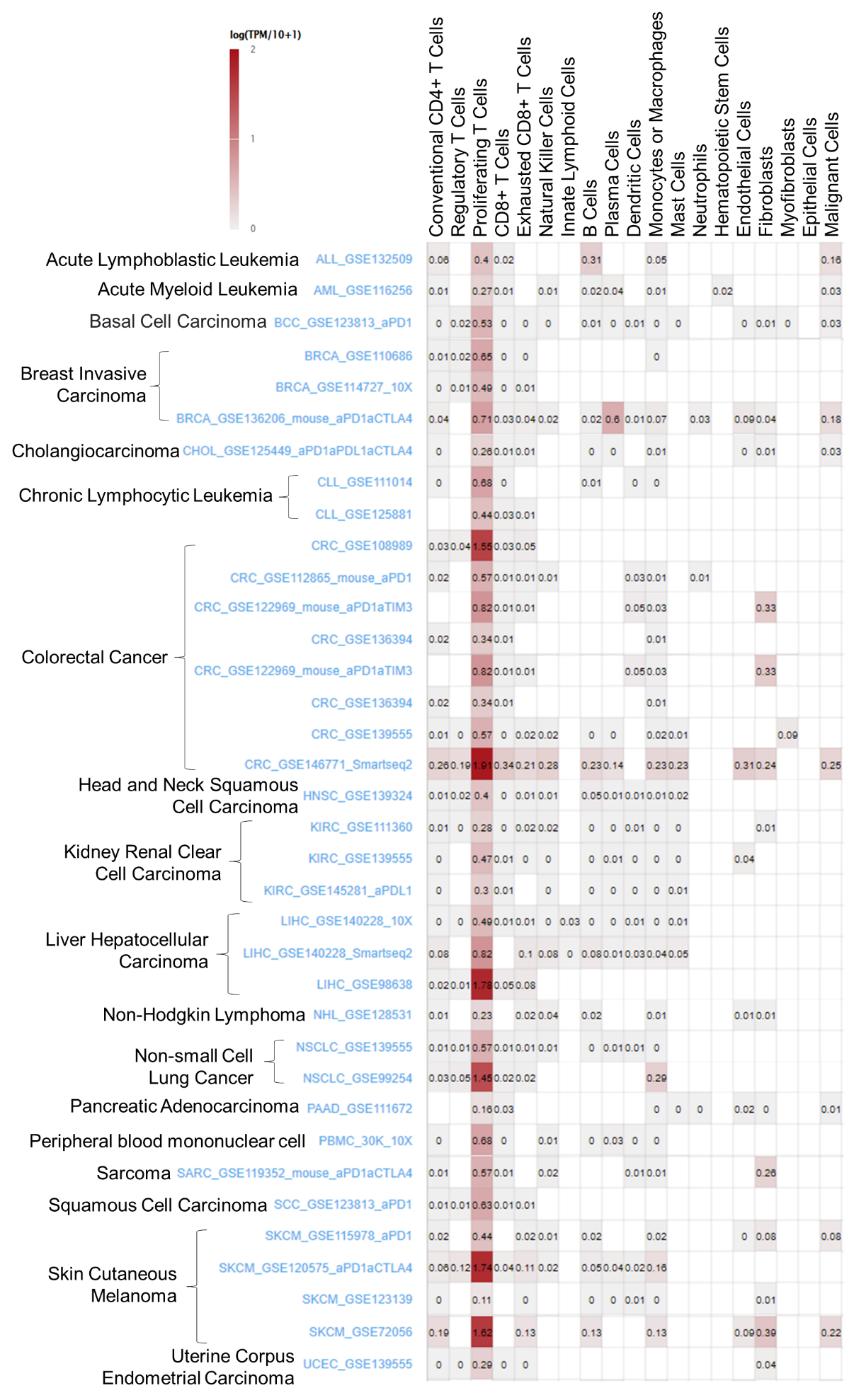


**S-Figure 5.** Heatmap of average RAD51 expression in different types of single cells in different cancer samples across multiple single-cell datasets. The single-cell data were accessed and analyzed using the TISCH.


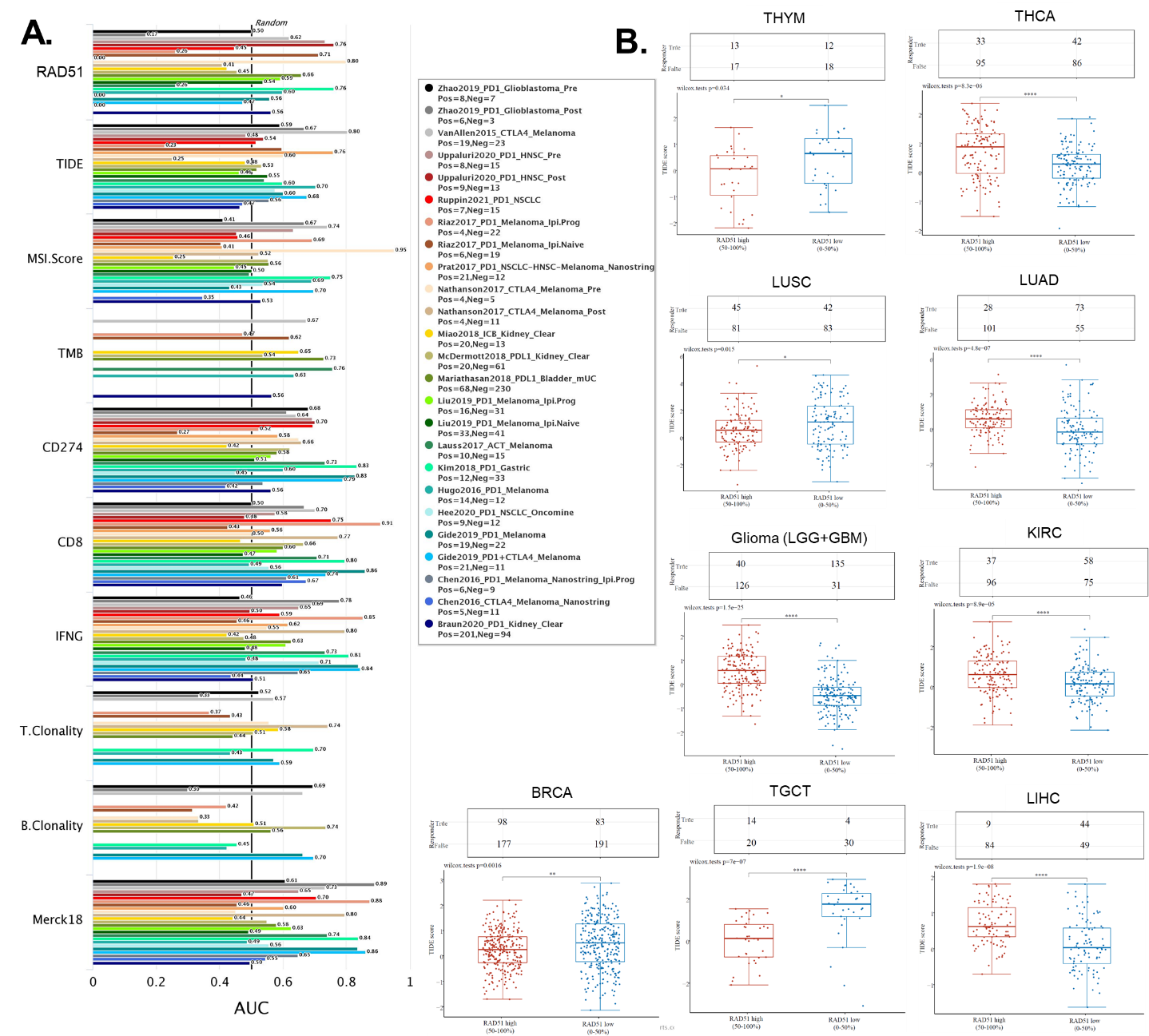


**S-Figure 6.** The predictive value of RAD51 for immune therapy in cancers. **A.** Bar plot showing the biomarker relevance of RAD51 compared to standardized cancer immune evasion biomarkers in immune checkpoint blockade (ICB) sub-cohorts. The area under the receiver operating characteristic curve (AUC) was applied to evaluate the predictive performances of the test biomarkers on the ICB response status. **B.** Immune checkpoint blockade (ICB) of RAD51 low (0-25%) and high (75-100%) groups were compared across multiple cancer types. Potential ICB response was predicted using the Tumor Immune Dysfunction and Exclusion (TIDE) algorithm. TCGA data were analyzed. Only significant cancer types were shown. (*P<0.05; **P<0.01; ***P<0.001; ****P<0.0001).


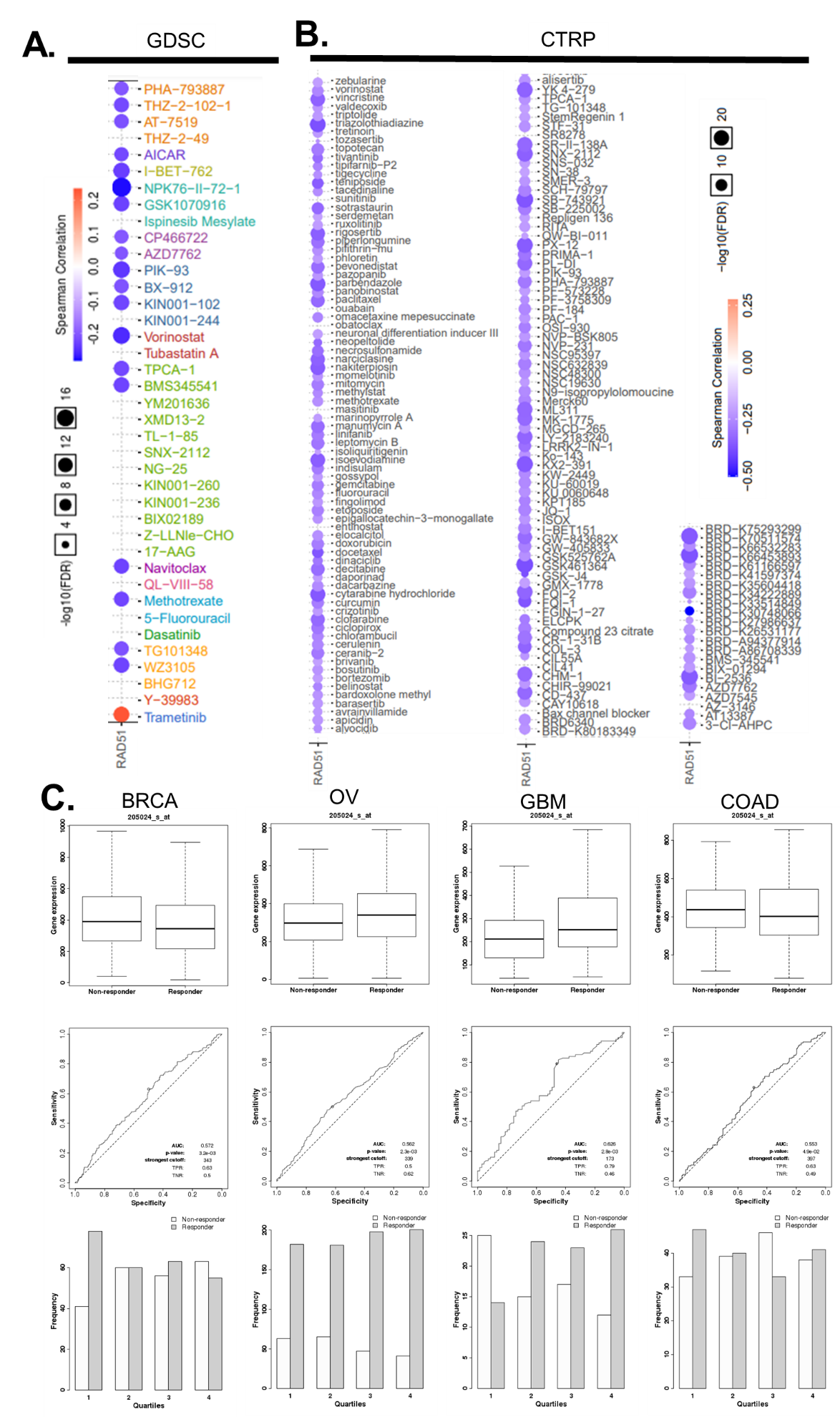


**S-Figure 7.** The predictive value of RAD51 for drug therapy in cancers. **A.** GDSC drugs, **B.** CTRP drugs. Drug sensitivity and gene expression profiling data of cancer cell lines in GDSC and CTRP are integrated for investigation. The expression of RAD51 was performed by Spearman correlation analysis with the small molecule/drug sensitivity (IC50). **C.** The expression of RAD51 in responder and nonresponder, chemotherapy predictive ROC plot, and chemotherapy response in RAD51 quartiles expression groups in BRCA, OV, GBM (female), and COAD (non-chemotherapy) cohorts. Data were accessed and analyzed using the ROC Plotter.


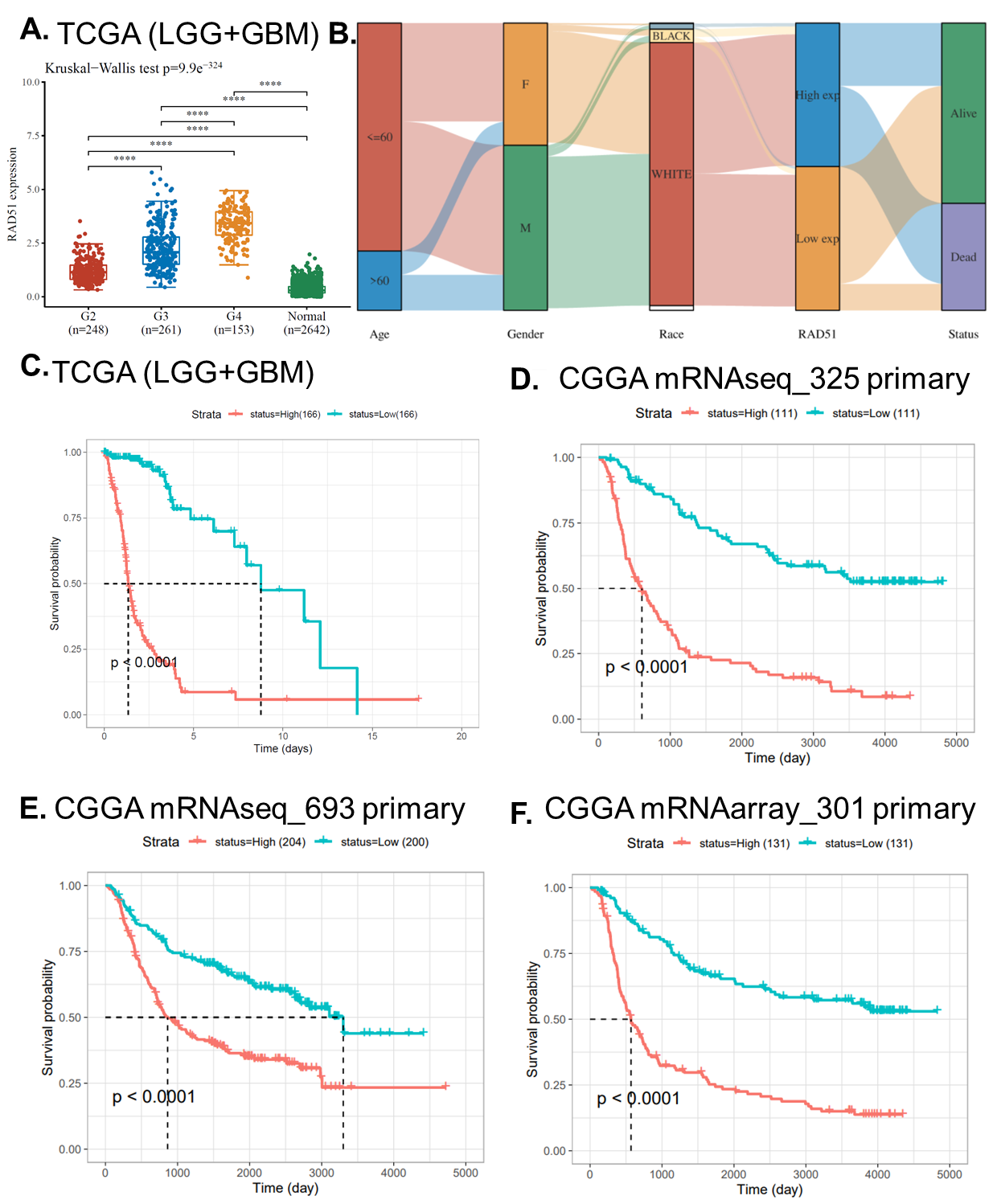


**S-Figure 8.** RAD51 in overall glioma. **A.** The expression of RAD51 in different types of cells in glioma samples. The single-cell data were accessed and analyzed using TISCH. **B.** Sankey diagram of overall glioma data from TCGA (LGG+GBM). **C.** Survival association of RAD51 in overall glioma patients using TCGA (LGG+GBM) data (discovered cohort). **D-F.** External validation of the survival association of RAD51**.** KM plot and Log-rank analysis of RAD51 in primary glioma of CGGA-mRNAseq_693 (validation cohort 1), CGGA-mRNAseq_325 (validation cohort 2), and CGGA- mRNA-array_301 (validation cohort 3).

**S-Table 1.** List of the cancer type abbreviations.

| Abbreviations | Full names |
| --- | --- |
| ACC | Adrenocortical carcinoma |
| BLCA | Bladder Urothelial Carcinoma |
| BRCA | Breast invasive carcinoma |
| CESC | Cervical squamous cell carcinoma and endocervical adenocarcinoma |
| CHOL | Cholangio carcinoma |
| COAD | Colon adenocarcinoma |
| DLBC | Lymphoid Neoplasm Diffuse Large B-cell Lymphoma |
| ESCA | Esophageal carcinoma |
| GBM | Glioblastoma multiforme |
| HNSC | Head and Neck squamous cell carcinoma |
| KICH | Kidney Chromophobe |
| KIRC | Kidney renal clear cell carcinoma |
| KIRP | Kidney renal papillary cell carcinoma |
| LAML | Acute Myeloid Leukemia |
| LGG | Brain Lower Grade Glioma |
| LIHC | Liver hepatocellular carcinoma |
| LUAD | Lung adenocarcinoma |
| LUSC | Lung squamous cell carcinoma |
| MESO | Mesothelioma |
| OV | Ovarian serous cystadenocarcinoma |
| PAAD | Pancreatic adenocarcinoma |
| PCPG | Pheochromocytoma and Paraganglioma |
| PRAD | Prostate adenocarcinoma |
| READ | Rectum adenocarcinoma |
| SARC | Sarcoma |
| SKCM | Skin Cutaneous Melanoma |
| STAD | Stomach adenocarcinoma |
| TGCT | Testicular Germ Cell Tumors |
| THCA | Thyroid carcinoma |
| THYM | Thymoma |
| UCEC | Uterine Corpus Endometrial Carcinoma |
| UCS | Uterine Carcinosarcoma |
| UVM | Uveal Melanoma |

**S-Table 2.** Top 100 correlated expressing genes to RAD51.

| Gene Symbol | Gene ID | Pearson correlation coefficient |
| --- | --- | --- |
| [OIP5](http://gepia.cancer-pku.cn/detail.php?gene=OIP5) | ENSG00000104147.8 | 0.88 |
| [CCNB2](http://gepia.cancer-pku.cn/detail.php?gene=CCNB2) | ENSG00000157456.7 | 0.84 |
| [CDC25A](http://gepia.cancer-pku.cn/detail.php?gene=CDC25A) | ENSG00000164045.11 | 0.83 |
| [CDCA5](http://gepia.cancer-pku.cn/detail.php?gene=CDCA5) | ENSG00000146670.9 | 0.81 |
| [ORC6](http://gepia.cancer-pku.cn/detail.php?gene=ORC6) | ENSG00000091651.8 | 0.81 |
| [DLGAP5](http://gepia.cancer-pku.cn/detail.php?gene=DLGAP5) | ENSG00000126787.12 | 0.8 |
| [ERCC6L](http://gepia.cancer-pku.cn/detail.php?gene=ERCC6L) | ENSG00000186871.6 | 0.8 |
| [FANCD2](http://gepia.cancer-pku.cn/detail.php?gene=FANCD2) | ENSG00000144554.10 | 0.8 |
| [NCAPH](http://gepia.cancer-pku.cn/detail.php?gene=NCAPH) | ENSG00000121152.9 | 0.8 |
| [BUB1B](http://gepia.cancer-pku.cn/detail.php?gene=BUB1B) | ENSG00000156970.12 | 0.79 |
| [CENPA](http://gepia.cancer-pku.cn/detail.php?gene=CENPA) | ENSG00000115163.14 | 0.78 |
| [EXO1](http://gepia.cancer-pku.cn/detail.php?gene=EXO1) | ENSG00000174371.16 | 0.78 |
| [HJURP](http://gepia.cancer-pku.cn/detail.php?gene=HJURP) | ENSG00000123485.11 | 0.78 |
| [MIS18A](http://gepia.cancer-pku.cn/detail.php?gene=MIS18A) | ENSG00000159055.3 | 0.78 |
| [RACGAP1](http://gepia.cancer-pku.cn/detail.php?gene=RACGAP1) | ENSG00000161800.12 | 0.78 |
| [ZWILCH](http://gepia.cancer-pku.cn/detail.php?gene=ZWILCH) | ENSG00000174442.11 | 0.78 |
| [CENPH](http://gepia.cancer-pku.cn/detail.php?gene=CENPH) | ENSG00000153044.9 | 0.77 |
| [FANCI](http://gepia.cancer-pku.cn/detail.php?gene=FANCI) | ENSG00000140525.17 | 0.77 |
| [TTK](http://gepia.cancer-pku.cn/detail.php?gene=TTK) | ENSG00000112742.9 | 0.77 |
| [AUNIP](http://gepia.cancer-pku.cn/detail.php?gene=AUNIP) | ENSG00000127423.10 | 0.76 |
| [DTL](http://gepia.cancer-pku.cn/detail.php?gene=DTL) | ENSG00000143476.17 | 0.76 |
| [NUSAP1](http://gepia.cancer-pku.cn/detail.php?gene=NUSAP1) | ENSG00000137804.12 | 0.76 |
| [PLK1](http://gepia.cancer-pku.cn/detail.php?gene=PLK1) | ENSG00000166851.14 | 0.76 |
| [RAD54L](http://gepia.cancer-pku.cn/detail.php?gene=RAD54L) | ENSG00000085999.11 | 0.76 |
| [SGOL1](http://gepia.cancer-pku.cn/detail.php?gene=SGOL1) | ENSG00000129810.14 | 0.76 |
| [BUB1](http://gepia.cancer-pku.cn/detail.php?gene=BUB1) | ENSG00000169679.14 | 0.75 |
| [C17orf53](http://gepia.cancer-pku.cn/detail.php?gene=C17orf53) | ENSG00000125319.14 | 0.75 |
| [CCNB1](http://gepia.cancer-pku.cn/detail.php?gene=CCNB1) | ENSG00000134057.14 | 0.75 |
| [CCNF](http://gepia.cancer-pku.cn/detail.php?gene=CCNF) | ENSG00000162063.12 | 0.75 |
| [KIAA1524](http://gepia.cancer-pku.cn/detail.php?gene=KIAA1524) | ENSG00000163507.13 | 0.75 |
| [KIF23](http://gepia.cancer-pku.cn/detail.php?gene=KIF23) | ENSG00000137807.13 | 0.75 |
| [KPNA2](http://gepia.cancer-pku.cn/detail.php?gene=KPNA2) | ENSG00000182481.8 | 0.75 |
| [MTFR2](http://gepia.cancer-pku.cn/detail.php?gene=MTFR2) | ENSG00000146410.11 | 0.75 |
| [MYBL2](http://gepia.cancer-pku.cn/detail.php?gene=MYBL2) | ENSG00000101057.15 | 0.75 |
| [NCAPG](http://gepia.cancer-pku.cn/detail.php?gene=NCAPG) | ENSG00000109805.9 | 0.75 |
| [PLK4](http://gepia.cancer-pku.cn/detail.php?gene=PLK4) | ENSG00000142731.10 | 0.75 |
| [PRC1](http://gepia.cancer-pku.cn/detail.php?gene=PRC1) | ENSG00000198901.13 | 0.75 |
| [SKA1](http://gepia.cancer-pku.cn/detail.php?gene=SKA1) | ENSG00000154839.9 | 0.75 |
| [CHEK1](http://gepia.cancer-pku.cn/detail.php?gene=CHEK1) | ENSG00000149554.12 | 0.74 |
| [MND1](http://gepia.cancer-pku.cn/detail.php?gene=MND1) | ENSG00000121211.7 | 0.74 |
| [RFWD3](http://gepia.cancer-pku.cn/detail.php?gene=RFWD3) | ENSG00000168411.13 | 0.74 |
| [SHCBP1](http://gepia.cancer-pku.cn/detail.php?gene=SHCBP1) | ENSG00000171241.8 | 0.74 |
| [TIMELESS](http://gepia.cancer-pku.cn/detail.php?gene=TIMELESS) | ENSG00000111602.11 | 0.74 |
| [CDCA3](http://gepia.cancer-pku.cn/detail.php?gene=CDCA3) | ENSG00000111665.11 | 0.73 |
| [KIF11](http://gepia.cancer-pku.cn/detail.php?gene=KIF11) | ENSG00000138160.5 | 0.73 |
| [KIF2C](http://gepia.cancer-pku.cn/detail.php?gene=KIF2C) | ENSG00000142945.12 | 0.73 |
| [KIFC1](http://gepia.cancer-pku.cn/detail.php?gene=KIFC1) | ENSG00000237649.7 | 0.73 |
| [MCM6](http://gepia.cancer-pku.cn/detail.php?gene=MCM6) | ENSG00000076003.4 | 0.73 |
| [ORC1](http://gepia.cancer-pku.cn/detail.php?gene=ORC1) | ENSG00000085840.12 | 0.73 |
| [POLR2D](http://gepia.cancer-pku.cn/detail.php?gene=POLR2D) | ENSG00000144231.10 | 0.73 |
| [TICRR](http://gepia.cancer-pku.cn/detail.php?gene=TICRR) | ENSG00000140534.13 | 0.73 |
| [TRAIP](http://gepia.cancer-pku.cn/detail.php?gene=TRAIP) | ENSG00000183763.8 | 0.73 |
| [XRCC2](http://gepia.cancer-pku.cn/detail.php?gene=XRCC2) | ENSG00000196584.2 | 0.73 |
| [ZWINT](http://gepia.cancer-pku.cn/detail.php?gene=ZWINT) | ENSG00000122952.16 | 0.73 |
| [CCNA2](http://gepia.cancer-pku.cn/detail.php?gene=CCNA2) | ENSG00000145386.9 | 0.72 |
| [CENPO](http://gepia.cancer-pku.cn/detail.php?gene=CENPO) | ENSG00000138092.10 | 0.72 |
| [GSG2](http://gepia.cancer-pku.cn/detail.php?gene=GSG2) | ENSG00000177602.5 | 0.72 |
| [GTSE1](http://gepia.cancer-pku.cn/detail.php?gene=GTSE1) | ENSG00000075218.18 | 0.72 |
| [KIF14](http://gepia.cancer-pku.cn/detail.php?gene=KIF14) | ENSG00000118193.11 | 0.72 |
| [MSH2](http://gepia.cancer-pku.cn/detail.php?gene=MSH2) | ENSG00000095002.12 | 0.72 |
| [PRIM1](http://gepia.cancer-pku.cn/detail.php?gene=PRIM1) | ENSG00000198056.13 | 0.72 |
| [ARHGAP11A](http://gepia.cancer-pku.cn/detail.php?gene=ARHGAP11A) | ENSG00000198826.10 | 0.71 |
| [CASC5](http://gepia.cancer-pku.cn/detail.php?gene=CASC5) | ENSG00000137812.19 | 0.71 |
| [CDC25C](http://gepia.cancer-pku.cn/detail.php?gene=CDC25C) | ENSG00000158402.18 | 0.71 |
| [CDT1](http://gepia.cancer-pku.cn/detail.php?gene=CDT1) | ENSG00000167513.8 | 0.71 |
| [DBF4](http://gepia.cancer-pku.cn/detail.php?gene=DBF4) | ENSG00000006634.7 | 0.71 |
| [DSCC1](http://gepia.cancer-pku.cn/detail.php?gene=DSCC1) | ENSG00000136982.5 | 0.71 |
| [MCM10](http://gepia.cancer-pku.cn/detail.php?gene=MCM10) | ENSG00000065328.16 | 0.71 |
| [NCAPG2](http://gepia.cancer-pku.cn/detail.php?gene=NCAPG2) | ENSG00000146918.19 | 0.71 |
| [NUP37](http://gepia.cancer-pku.cn/detail.php?gene=NUP37) | ENSG00000075188.8 | 0.71 |
| [SPC25](http://gepia.cancer-pku.cn/detail.php?gene=SPC25) | ENSG00000152253.8 | 0.71 |
| [TIPIN](http://gepia.cancer-pku.cn/detail.php?gene=TIPIN) | ENSG00000075131.9 | 0.71 |
| [TROAP](http://gepia.cancer-pku.cn/detail.php?gene=TROAP) | ENSG00000135451.12 | 0.71 |
| [UBE2T](http://gepia.cancer-pku.cn/detail.php?gene=UBE2T) | ENSG00000077152.9 | 0.71 |
| [C16orf59](http://gepia.cancer-pku.cn/detail.php?gene=C16orf59) | ENSG00000162062.14 | 0.7 |
| [CDCA8](http://gepia.cancer-pku.cn/detail.php?gene=CDCA8) | ENSG00000134690.10 | 0.7 |
| [CHAF1A](http://gepia.cancer-pku.cn/detail.php?gene=CHAF1A) | ENSG00000167670.15 | 0.7 |
| [FEN1](http://gepia.cancer-pku.cn/detail.php?gene=FEN1) | ENSG00000168496.3 | 0.7 |
| [KIF15](http://gepia.cancer-pku.cn/detail.php?gene=KIF15) | ENSG00000163808.16 | 0.7 |
| [KIF18A](http://gepia.cancer-pku.cn/detail.php?gene=KIF18A) | ENSG00000121621.6 | 0.7 |
| [MCM4](http://gepia.cancer-pku.cn/detail.php?gene=MCM4) | ENSG00000104738.16 | 0.7 |
| [RAD51AP1](http://gepia.cancer-pku.cn/detail.php?gene=RAD51AP1) | ENSG00000111247.14 | 0.7 |
| [RANBP1](http://gepia.cancer-pku.cn/detail.php?gene=RANBP1) | ENSG00000099901.16 | 0.7 |
| [TDP1](http://gepia.cancer-pku.cn/detail.php?gene=TDP1) | ENSG00000042088.13 | 0.7 |
| [BRCA1](http://gepia.cancer-pku.cn/detail.php?gene=BRCA1) | ENSG00000012048.19 | 0.69 |
| [CDC45](http://gepia.cancer-pku.cn/detail.php?gene=CDC45) | ENSG00000093009.9 | 0.69 |
| [CKAP2L](http://gepia.cancer-pku.cn/detail.php?gene=CKAP2L) | ENSG00000169607.12 | 0.69 |
| [CLSPN](http://gepia.cancer-pku.cn/detail.php?gene=CLSPN) | ENSG00000092853.13 | 0.69 |
| [EME1](http://gepia.cancer-pku.cn/detail.php?gene=EME1) | ENSG00000154920.14 | 0.69 |
| [EZH2](http://gepia.cancer-pku.cn/detail.php?gene=EZH2) | ENSG00000106462.10 | 0.69 |
| [GINS1](http://gepia.cancer-pku.cn/detail.php?gene=GINS1) | ENSG00000101003.9 | 0.69 |
| [HELLS](http://gepia.cancer-pku.cn/detail.php?gene=HELLS) | ENSG00000119969.14 | 0.69 |
| [KIF20A](http://gepia.cancer-pku.cn/detail.php?gene=KIF20A) | ENSG00000112984.11 | 0.69 |
| [MCM5](http://gepia.cancer-pku.cn/detail.php?gene=MCM5) | ENSG00000100297.15 | 0.69 |
| [NASP](http://gepia.cancer-pku.cn/detail.php?gene=NASP) | ENSG00000132780.16 | 0.69 |
| [NEK2](http://gepia.cancer-pku.cn/detail.php?gene=NEK2) | ENSG00000117650.12 | 0.69 |
| [NUF2](http://gepia.cancer-pku.cn/detail.php?gene=NUF2) | ENSG00000143228.12 | 0.69 |
| [PIF1](http://gepia.cancer-pku.cn/detail.php?gene=PIF1) | ENSG00000140451.12 | 0.69 |
| [POLE2](http://gepia.cancer-pku.cn/detail.php?gene=POLE2) | ENSG00000100479.12 | 0.69 |
| [TPX2](http://gepia.cancer-pku.cn/detail.php?gene=TPX2) | ENSG00000088325.15 | 0.69 |
